# Dynamic Interplay of Host and Pathogens in an Avian Whole-Blood Model

**DOI:** 10.3389/fimmu.2020.00500

**Published:** 2020-03-31

**Authors:** Sravya Sreekantapuram, Teresa Lehnert, Maria T. E. Prauße, Angela Berndt, Christian Berens, Marc Thilo Figge, Ilse D. Jacobsen

**Affiliations:** ^1^Research Group Microbial Immunology, Leibniz Institute for Natural Product Research and Infection Biology, Hans Knöll Institut, Jena, Germany; ^2^Research Group Applied Systems Biology, Leibniz Institute for Natural Product Research and Infection Biology, Hans Knöll Institut, Jena, Germany; ^3^Faculty of Biological Sciences, Institute of Microbiology, Friedrich Schiller University Jena, Jena, Germany; ^4^Institute of Molecular Pathogenesis, Friedrich-Loeffler-Institut, Jena, Germany

**Keywords:** chicken whole blood, avian immune response, *Escherichia coli*, *Staphylococcus aureus*, *Candida albicans*

## Abstract

Microbial survival in blood is an essential step toward the development of disseminated diseases and blood stream infections. For poultry, however, little is known about the interactions of host cells and pathogens in blood. We established an *ex vivo* chicken whole-blood infection assay as a tool to analyze interactions between host cells and three model pathogens, *Escherichia coli, Staphylococcus aureus*, and *Candida albicans*. Following a systems biology approach, we complemented the experimental measurements with functional and quantitative immune characteristics by virtual infection modeling. All three pathogens were killed in whole blood, but each to a different extent and with different kinetics. Monocytes, and to a lesser extent heterophils, associated with pathogens. Both association with host cells and transcriptional activation of genes encoding immune-associated functions differed depending on both the pathogen and the genetic background of the chickens. Our results provide first insights into quantitative interactions of three model pathogens with different immune cell populations in avian blood, demonstrating a broad spectrum of different characteristics during the immune response that depends on the pathogen and the chicken line.

## Introduction

Bacterial infections in chicken affect not only animal health and welfare, but also have significant economic impact (1), due to the increasing restrictions in the use of antimicrobials in order to prevent the increase of antibiotic resistance in zoonotic bacteria. Since the emergence of resistant bacteria might impair the efficacy of antibiotic treatment, alternative approaches to combating bacterial infections in poultry are necessary. One possibility is the development of vaccines (2), another selective breeding aimed at higher intrinsic resistance (3). For a rational approach to either of the strategies, it is however necessary to understand the host response to the infection. While the avian response to zoonotic *Salmonella* and *Campylobacter* has been studied in detail, the knowledge on the response of the avian immune system to other bacterial pathogens is very limited (4).

This applies for example to colibacillosis, an infection caused by pathogenic strains of the Gram-negative bacterium *Escherichia coli*. Colibacillosis often initially manifests in the respiratory tract, but the bacteria can spread into the blood stream leading to colisepticaemia and infection of distal body sites and organs (5, 6). Survival in the blood stream is an essential feature of *E. coli* strains to be able to cause disseminated colibacillosis, as exemplified by the correlation of serum resistance and the ability to survive in the blood stream and infect internal organs in chickens (7, 8). While the recruitment of immune cells to solid organs (9), and the transcriptional response of internal organs and peripheral blood leukocytes to colibacillosis has been studied (10, 11), to our knowledge, it remains unknown which immune cells interact with *E. coli* within avian blood.

Another common agent causing infections in poultry is *Staphylococcus aureus*, a Gram-positive bacterium (12, 13). *S. aureus* infections can affect various organ systems, including skin, mucosal membranes, and, via hematogenous spread, also tendon sheaths, joints, and bones (12, 14, 15). In severe cases, septicemia occurs (13). As for infections with *E. coli*, the immune response of chickens to *S. aureus* has not been studied in detail and it remains unclear which immune cells interact with these bacteria in blood during dissemination or septicemia. In order to investigate the interactions with immune cells and the fate of the pathogens in avian blood, we adapted a human whole-blood infection assay previously described for analyzing interactions between blood components and the facultative fungal pathogen *Candida albicans* (16, 17) and the host response to bacterial infection (18). *C. albicans* was also included in this study; it is a common colonizer of mucosal surfaces of a variety of birds, including chickens, but also one of the most frequent causes of fungal infection (19, 20). Infections predominantly affect the mucosa of the crop, esophagus and intestine, but hematogenous dissemination can occur, leading to retarded growth, hepatic and renal congestion, and neural disturbance (19, 21, 22).

Because it had previously been shown that host genetics can have a significant influence on infections in chickens (23, 24), two different White Leghorn chicken lines were used. These lines (WLA and R11) differ in their egg laying performance (25), susceptibility to lipopolysaccharide (LPS) challenge (26), and response to avian influenza virus (27).

In line with our previous studies on whole-blood infections in humans (17, 28–30), the experimental whole-blood infection assay was complemented by virtual infection modeling. By calibrating the virtual infection model to experimental data, the functional characteristics of the immune response in avian whole blood were quantified. Moreover, representing the complexity of whole-blood by a mechanistic mathematical model enabled us to identify essential and novel immune processes during the immune response in avian whole blood. To this end, we implemented several state-based virtual infection models that differ by the presence of potential immune response mechanisms, like the killing of pathogens in extracellular space or by immune cells.

## Materials and Methods

### Animals and Ethics Statement

Two White Leghorn chicken lines differing in their egg laying performances were used in this study: WLA as a high producing line and R11, a low producer (25, 26, 31). WLA originates from a breeding line of Lohmann Tierzucht GmbH, Cuxhaven. The White Leghorn line R11 has been managed as conservation flock at the institute since 1965, originally derived from the Cornell Line K (32). Chicks were hatched from the eggs (kindly provided by Prof. Steffen Weigend; ING) and housed at the facilities of Friedrich-Loeffler-Institut, Jena, Germany under pathogen free conditions. Animal housing was performed in accordance with the guidelines for animal welfare set by the European Community. Throughout the study, the chickens were reared and kept under standardized conditions at 18–20°C and a relative humidity of 50–60%. Commercial feed in powder form (without antibiotics or other additives) and drinking water were both available *ad libitum*. The study was carried out in strict accordance with the German Animal Welfare Act under supervision of the authorized institutional Agent for Animal Protection. Six animals, aged 16–19 months, from each chicken line were used for each experiment conducted in this study.

### Whole-Blood *ex-vivo* Infection Assay

Blood samples (total amount: four ml per animal and sampling) were collected by jugular venipuncture into commercial hirudin-coated syringes (S-Monovette®, 2.7 ml Hirudin, Sarstedt, Germany). Hirudin was chosen as anti-coagulant as it was previously shown to have no effect on complement activation (33). After addition of 10^6^ microbial cells per ml, the blood was incubated at 40^o^C, 5% CO_2_ under constant rotation for 240 min. Samples were collected every 30 min for flow cytometry, microscopy, determination of colony forming units (CFU), and PCR.

### Pathogens Used in This Study

GFP-expressing strains of *Candida albicans* [CaGFP (17)], *Staphylococcus aureus* [6850/pALC1743 (34, 35)] and *Escherichia coli* ATCC 25922 (36) were used for *ex vivo* infection of avian blood. To generate the GFP-expressing *E. coli*, a plasmid constitutively expressing the GFP-variant GFP+ (37) was constructed by first fusing the promoter of the *cat* gene from pACYC184 (38) to the coding sequence of the *gfp*+ gene via overlap extension PCR (39) using a thermostable high-fidelity DNA polymerase and the following oligonucleotides: PcatXbaI-F: 5′-CATGAATCTAGAACGGAAGATCACTTCGCAG-3′; CatSDGFP-R: 5′-CTTCTCCTTTGCTAGCCATTTTAGCTCCTCCTCGATAACTCAAAAAATACGCC-3′; CatSDGFP-F: 5′-GGCGTATTTTTTGAGTTATCGAGGAGGAGCTAAAATGGCTAGCAAAGGAGAAG-3′.

GFP+EcoRI-R: 5′-ACCAACTGGTAATGGTAGC-3′. The resulting 923 bp PCR fragment was restricted with EcoRI and XbaI (New England Biolabs, Frankfurt, Germany) and ligated with likewise-restricted pUC19 (40) yielding the plasmid pUC19Pcatgfp+. Chemically competent cells of *E. coli* ATCC 25922 (36) were then transformed with the plasmid pUC19Pcatgfp+.

*C. albicans* was cultivated overnight in yeast extract peptone dextrose (YPD) medium (20 g/l peptone, Otto Nordwald, Hamburg, Germany; 10 g/l yeast extract, Serva, Heidelberg, Germany; 20 g/l dextrose, Carl Roth, Karlsruhe, Germany; pH adjusted to 7.0 with NaOH) at 30^o^C, 180 rpm. The overnight culture was inoculated 1:50 into fresh YPD medium and incubated at 30^o^C, 180 rpm until OD_600_ 1.0 was reached. *S. aureus* and *E. coli* were cultivated overnight at 37^o^C, 180 rpm in lysogeny broth (LB medium: tryptone 10 g/l, Carl Roth; yeast extract 5 g/l, Serva; sodium chloride 10 g/l, Carl Roth; pH adjusted to 7.0 with NaOH). The overnight culture was inoculated 1:100 into fresh LB medium and incubated at 37°C, 180 rpm until OD_600_ 0.6–0.7 was reached. The cultures were then washed thrice in phosphate buffered saline (PBS). The number of *C. albicans* cells was determined by counting using a Neubauer chamber. The number of bacterial cells was calculated based on the OD_600_ - CFU correlation. Cultures were diluted to the desired concentrations with PBS before inoculation of whole blood.

### Quantification of Pathogen Survival

To determine the survival of pathogens in avian whole blood, serial dilutions of the samples collected at different time points were plated on blood agar plates in 2–4 technical replicates. CFU counts were determined after overnight incubation at 30°C (for *C. albicans*) and 37°C (for *S. aureus* and *E. coli*).

### Flow Cytometry

To determine both the number of immune cells and which immune cells interacted with the pathogens, cells were incubated with monoclonal antibodies (mAB) targeting the monocyte/macrophage marker KULO1-RPE (41), the macrophage/thrombocyte marker K1-RPE (42), the leucocyte marker CD45-APC (43), the B-cell marker BU1-APC-Cy7, and the T-cell marker CD3-SPRD (PE-Cy5) (44, 45). All antibodies were obtained from Southern biotechnology associates (Eching, Germany). Conjugation of mAb K1 to R-PE and BU1 to APC-Cy7 was performed using the respective Abcam conjugation kits according to the manufacturer's instructions. For staining, 50 μl of blood diluted 1:50 with PBS was mixed with 20 μl of an antibody mixture containing the directly conjugated K1, KULO1, CD45, BU1, and CD3 in the end concentrations of 0.2 μg in a Trucount tube containing beads for absolute quantification (BD Biosciences; Heidelberg, Germany) and incubated at room temperature for 45 min in the dark. 300 μl of PBS was then added to the sample, which was kept in the dark until measurement. The measurements were performed on a FACSCanto II (BD Bioscences; Heidelberg, Germany) and analyzed using the software FACSDiva (Version 6.1.3, BD Biosciences). Up to 20,000 trucount beads were recorded together with immune cells in each sample, for absolute quantification of the cell populations. Absolute cell numbers were then calculated using the following formula (46):

(1) $$\begin{array}{l} \frac{Absolute\;cell\;count\;}{\mu l\;of\;blood\;}=\frac{cells\;counted}{beads\;counted}\\ \;\times \frac{total\;content\;of\;beads\;per\;tube}{blood\;volume\;per\;tube}. \end{array}$$

Numbers of monocytes and thrombocytes were calculated from the dot plot K1/KULO1 against CD45. Single populations of T and B cells were obtained from the CD45^+^ but K1^−^/KUL01^−^ leucocyte population shown in the dot plot of CD3 against BU1. Heterophils were identified within the CD45^+^ cell population plotted against SSC. All single populations were back-gated against FSC/SSC for their absolute number calculations. Prior to analysis of immune cell populations, doublets were excluded by means of the FSC-H and FSC-A dot pot. The GFP-expressing pathogens were identified and recorded using the FITC channel and were sub-gated against immune cell specific markers to obtain the percentage of pathogens interacting with the different immune cells.

### RNA Extraction and Quantitative Real-Time Reverse Transcription (RT)-PCR

To analyze the transcription of immune-related genes, total RNA was extracted from 100 μl of blood using the RNeasy Mini Kit for blood (Qiagen) according to the manufacturer's protocol. The QuantiTect SYBR Green real-time one-step RT-PCR kit (Qiagen) and avian-specific primers for IFN-γ, IL-1β, IL-6, IL-8, iNOS, K60, LITAF and MIP-1β (47–49) were used to determine mRNA expression levels. The expression was normalized to the house keeping gene glycerinaldehyde-3-phosphat (GAPDH) and expressed as fold change compared to non-infected samples using the threshold method for quantification (2^(−ΔΔC^_*T*_^)^) (50). Additional information on primer efficacy and *Ct* values for the housekeeping gene are provided in the Supplementary Material.

### Statistical Analyses

Six independent biological replicates derived from different animals were used for all experiments. Data is represented as arithmetic mean ± SD. Normality distribution was assessed using the Kolmogorov Smirnov test in GraphPad Prism 7. Data was analyzed by 2-way ANOVA followed by Tukey's multiple comparison test (GraphPad Prism 7) to compare infected and non-infected samples, different time points, different pathogens and different chicken lines. *P*-values < 0.05 were considered significant.

### Mathematical Modeling

**Figure 1:**
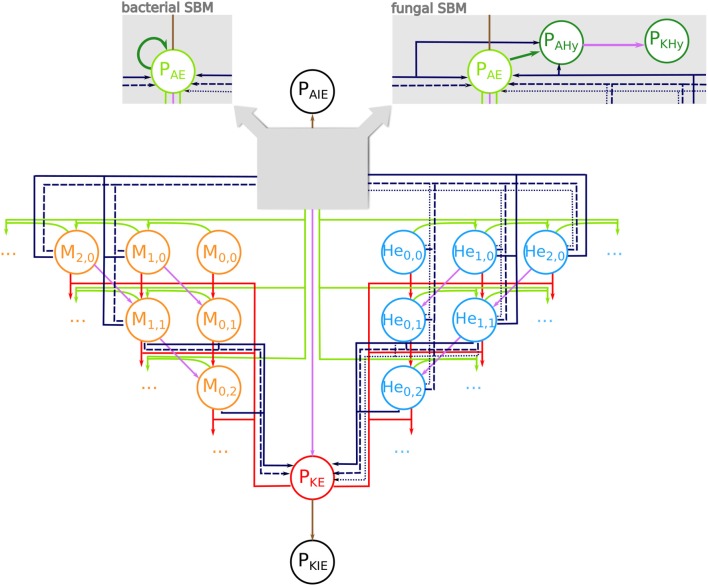
State-based model of avian whole-blood infection. Schematic picture of the state-based model (SBM) for the immune response in avian whole blood upon infection with either of the three pathogens *S. aureus* and *E. coli* or *C. albicans*. The states (circles) represent the different cell populations of pathogens (P) and the two immune cell types of monocytes (*M*_*i,j*_) and heterophils (*He*_*i,j*_) with *i* alive and *j* killed pathogens. The model contains respective states for extracellular pathogens that are alive (*P*_*AE*_) or killed (*P*_*KE*_) as well as immune evasive pathogens that are alive (*P*_*AIE*_) or have been killed (*P*_*KIE*_). The SBM for fungal infection additionally contains states for alive pathogens in hyphal form (*P*_*AHy*_) that can be killed by extracellular factors (*P*_*KHy*_) (see right gray box). In the bacterial SBM, alive extracellular pathogens can proliferate (dark green arrow) (see left gray box). Connections between the states refer to possible state transitions that represent biological reactions during infection. Alive and killed extracellular pathogens can be phagocytosed (green and red arrows). The purple connections indicate killing of pathogens either in extracellular space or within immune cells. The dark blue connections represent the different mechanisms of immune cell killing. These are heterophil killing by stress factors with rate $\kappa_{stress}^{He}$ (dotted lines), immune cell killing by extracellular peptides (dashed lines) and immune cell killing by lysis induced by alive intracellular pathogens (solid lines).

We adapted our human whole-blood model (17, 28–30) and generated state-based models (SBMs) that simulate the immune reactions during infection in avian whole-blood samples. In order to cope with known differences between fungal and bacterial infection scenarios in avian blood, we implemented slightly different models for bacteria and fungi. Both models comprise states that represent the various cell types, which take part in the immune response, i.e., heterophils (*He*), monocytes (*M*), and the pathogens (P). Furthermore, the SBMs contain states for different subpopulations of the pathogens, which are pathogens in extracellular space that are alive (*P*_*AE*_) or killed (*P*_*KE*_), and living or killed pathogens that are within the monocytes (*P*_*AM*_, *P*_*KM*_) or within the heterophils (*P*_*AHe*_, *P*_*KHe*_). Please note that living pathogens were termed “alive” and dead pathogens “killed” within the mathematical model, and that these phrases are used throughout this manuscript in this context. The number of alive and killed pathogens within in an immune cell is counted by the indizes *i* and *j*, respectively, so that monocytes and heterophils are represented by *M*_*i,j*_ and *He*_*i,j*_. Figure 1 depicts all states and possible state transitions of the SBMs for bacterial and fungal infection scenarios. The state transitions represent the immune reactions during infection with the pathogens. Since the knowledge about these reactions is very limited, we started with the human SBM as a basis and added reactions that are either known or mandatory to reconcile simulations with the experimental measurements.

This SBM contains nine transition rates that characterize the nine different reactions. In analogy to the human SBM, these include phagocytosis by monocytes (Φ_*M*_) and heterophils (Φ_*He*_), extracellular killing of pathogens ($\kappa_{EK}^{P}\left(t\right)$), intracellular killing of the pathogens in monocytes ($\kappa_{M}^{P}$) and in heterophils ($\kappa_{He}^{P}$) as well as a process where pathogens become immune evasive and can evade killing and/or phagocytosis (ρ). Furthermore, we added the killing of heterophils by stress factors that are independent of infection and induced by the experimental set up ($\kappa_{stress}^{He}$). Additionally, we assumed that in avian blood the monocytes and heterophils can be killed by a process caused by factors released by pathogens into the extracellular space ($\kappa_{EM}^{M}$, $\kappa_{EM}^{He})$. As previously indicated we implemented bacterial proliferation (*o*) and the hyphae formation of fungi (Ψ) as pathogen specific reactions. A complete list of the transition rates with the respective state transitions and a concise description is given in Table 1.

**Table 1:** Transition rates of the avian SBM. For details see Materials and Methods section and Hünniger et al. (17) and Lehnert et al. (28).

**Transition rate**	**Description**	**State transition**
ϕ_*M*_	Phagocytosis by monocytes	*M*_*i,j*_ + *P*_*AE*_ → *M*_*i* + 1, *j*_
ϕ_*He*_	Phagocytosis by heterophils	*He*_*i,j*_ + *P*_*AE*_ → *He*_*i* + 1, *j*_
$\kappa_{M}^{P}$	Intracellular killing of pathogens by monocytes	*M*_*i,j*_ → *M*_*i* − 1, *j* + 1_
$\kappa_{He}^{P}$	Intracellular killing of pathogens by heterophils	*He*_*i,j*_ → *He*_*i* − 1, *j* + 1_
$\kappa_{EK}^{P}\left(t\right)$	Extrallular killing by antimicrobial proteins that were released by first-time phagocytosis by heterophils. The rate depends on the activity of antimicrobial proteins ($\bar{\kappa_{EK}}$) and the decay of their activity (γ) as defined in Hünniger et al. (17) and Lehnert et al. (28)	*P*_*AE*_ → *P*_*KE*_*P*_*AHy*_ → *P*_*KHy*_
ρ	Acquire immune evasion against phagocytosis and/ or extracellular killing	*P*_*AE*_ → *P*_*AIE*_*P*_*KE*_ → *P*_*KIE*_
$\kappa_{stress}^{He}$	Killing of heterophils by stress	*He*_*i,j*_ → *iP*_*AE*_ + *jP*_*KE*_
$\kappa_{EM}^{M}$	Killing of monocytes by extracellular mechanisms that are pathogen dependent	*M*_*i,j*_ → *iP*_*AE*_ + *jP*_*KE*_
$\kappa_{EM}^{He}$	Killing of heterophils by mechanisms that are not cellularly induced but pathogen dependent	*He*_*i,j*_ → *iP*_*AE*_ + *jP*_*KE*_
*o*	Proliferation rate of bacteria	*P*_*AE*_ → 2*P*_*AE*_ *M*_*i,j*_ → *M*_*i* + 1, *j*_ *He*_*i,j*_ → *He*_*i* + 1, *j*_
Ψ	Formation of fungal hyphae in extracellular space	*P*_*AE*_ → *P*_*Hy*_
$\kappa_{EK}^{P}$	Extracellular killing by constant concentration of antimicrobials	*P*_*AE*_ → *P*_*KE*_ *P*_*AHy*_ → *P*_*KHy*_
$\kappa_{lysis}^{M}$	Killing of monocytes by pathogen-induced lysis	Fungal SBM: *M*_*i,j*_ → *P*_*Hy*_ + (*i* − 1)*P*_*AE*_ + *jP*_*KE*_ Bacterial SBM: *M*_*i,j*_ → *iP*_*AE*_ + *jP*_*KE*_
$\kappa_{lysis}^{He}$	Killing of heterophils by pathogen-induced lysis	*He*_*i,j*_ → *P*_*Hy*_ + (*i* − 1)*P*_*AE*_ + *jP*_*KE*_ *He*_*i,j*_ → *iP*_*AE*_ + *jP*_*KE*_

In order to take the morphological switch of *C. albicans* cells from yeast to hyphal form into account, the fungal SBM additionally contains states for alive pathogens in hyphal form (*P*_*AHy*_) and killed pathogens in hyphal form (*P*_*KHy*_). Furthermore, the fungal SBM comprises additional transitions that represent the switch to the hyphal form in extracellular space (*P*_*AE*_ → *P*_*AHy*_) with rate Ψ. Similar to alive extracellular *C. albicans* yeast cells (*P*_*AE*_), extracellular hyphae (*P*_*AHy*_) can also be killed (*P*_*AHy*_ → *P*_*KHy*_) by the same extracellular killing mechanisms as yeast. The SBM for the infection scenario with either of the bacteria *S. aureus* and *E. coli*, contains bacterial proliferation which takes place either within immune cells or in extracellular space with rate.

As mentioned before, the described SBMs for fungal and bacterial infection contain a single mechanism for killing of pathogens in extracellular space and, therefore, we refer to these models as SEK-SBMs (Single Extracellular Killing mechanism-SBMs). These SEK-SBMs differ only with respect to hyphae formation and proliferation in order to represent the differences between fungal and bacterial infection scenarios. In addition to the SEK-SBMs, we implemented the MEK-SBMs (Multiple Extracellular Killing mechanism-SBMs), where extracellular killing is not only caused by effectors released by immune cells upon first phagocytosis (with rate $\kappa_{EK}^{P}\left(t\right)$), but also caused by effectors present immediately upon infection. We assume that these effectors are present in high concentration so that their effect is temporally constant and does not decrease during the time of the infection. Therefore, we defined the constant rate $\kappa_{EK}^{P}$ for this transition in the MEK-SBMs (see Table 1).

In addition to immune cell killing by extracellular microbial factors with rate $\kappa_{EM}^{M}$ for monocytes and $\kappa_{EM}^{He}$ for heterophils, we considered the possibility that immune cells can be killed by intracellular pathogens. Here, we assumed that pathogens can escape phagocytosis by actively breaking through the immune cell membrane. Thereby, the immune cell membrane will be destroyed and alive and killed internalized pathogens will be released into the extracellular space. This lysis by pathogens takes place in monocytes with rate $\kappa_{lysis}^{M}\;$ and in heterophils with rate $\kappa_{lysis}^{He}$. The corresponding transitions are given in Table 1 for bacterial infections and for C*. albicans* infection, where this lytic escape is initiated by intracellular hyphae formation.

The SBMs were simulated by applying a random selection simulation algorithm (51), where the simulation time is divided into equidistant time intervals (Δ*t*) and at each discrete time step, each cell can perform a transition from state *S* to state *S*′ with probability $P_{S\to S^{\prime }}$ that is defined by $P_{S\to S^{\prime }}=r_{S\to S^{\prime }}\times \Delta t$. We used the simulation algorithm as described in form of a flow chart in (28). In order to compare the model simulation with the kinetics observed from experimental whole-blood infection assays, we defined so called combined units, which are composed of specific model states, in order to form the counterparts of the five experimental measurements. The survival assays yield the kinetics of alive and killed pathogens. In both models, the alive pathogens are combined in

(2) $$\begin{array}{l} P_{A}\equiv P_{AE}+P_{AIE}+\sum_{i\geq 0}\sum_{j\geq 0}\left(M_{i,j}+He_{i,j}\right)i \end{array}$$

In the SBM of the fungal infection scenario, the combined unit *P*_*A*_ additionally involves the alive pathogens in hyphal form *P*_*AHy*_. The killed pathogens in the models are summarized in

(3) $$\begin{array}{l} P_{K}\equiv P_{KE}+P_{KIE}+\sum_{i\geq 0}\sum_{j\geq 0}\left(M_{i,j}+He_{i,j}\right)j \end{array}$$

where again, the SBM of fungal infection scenario additionally involves *P*_*KHy*_, the killed pathogens in hyphal form. The measurements of the Flow Cytometry analysis, i.e., the association either to monocytes or heterophils, or to none of them were compared, respectively with the combined units

(4) $$\begin{array}{l} P_{M}\;\equiv \;\sum_{i\geq 0}\sum_{j\geq 0}\left(i+j\right)M_{i,j} \end{array}$$

(5) $$\begin{array}{l} P_{He}\;\equiv \;\sum_{i\geq 0}\sum_{j\geq 0}\left(i+j\right)He_{i,j} \end{array}$$

and

(6) $$\begin{array}{l} P_{E}\equiv P_{AE}+P_{KE}+P_{AIE}+P_{KIE} \end{array}$$

Note that the combined unit of pathogens in extracellular space, *P*_*E*_, also incorporates the alive and killed pathogens in hyphal form (*P*_*AHy*_, *P*_*KHy*_) in the fungal SBM, in comparison to the bacterial SBM. The total number of pathogens is given by *P* ≡ *P*_*E*_+*P*_*N*_+*P*_*M*_ and *P* ≡ *P*_*A*_+*P*_*K*_ in the fungal SBMs. In the bacterial SBMs *P* ≠ *P*_*A*_+*P*_*K*_, because the number of alive pathogens can increase by bacterial proliferation.

Since additionally the number of heterophils and monocytes could be quantified in the whole-blood assays, we defined combined units that, respectively, record the number of heterophils

(7) $$\begin{array}{l} He\;\equiv \;\sum_{i\geq 0}\sum_{j\geq 0}He_{i,j} \end{array}$$

and monocytes

(8) $$\begin{array}{l} M\;\equiv \;\sum_{i\geq 0}\sum_{j\geq 0}M_{i,j} \end{array}$$

Details on the parameter estimation procedure as well as on the comparison of various models by the Akaike information criterion are provided in the Supplementary Material.

## Results

### Chicken-Line Specific Decrease of Monocytes and Heterophils in Whole Blood

As the aim of this study was to analyze the interaction of pathogens with avian immune cells in an *ex vivo* whole-blood model, we first determined if the different leukocyte populations remained stable using flow cytometry. The absolute numbers of monocytes and T cells declined within the first 30–60 min for both chicken lines, but remained stable thereafter (Supplementary Figure 1, Supplementary Table 1). The number of B cells did not change over time. Thrombocytes moderately decreased within the first 60 min in blood from R11 chickens only, and heterophil numbers showed a slow steady decrease over time in WLA chickens (Supplementary Figure 1, Supplementary Table 1).

**Figure 2:**
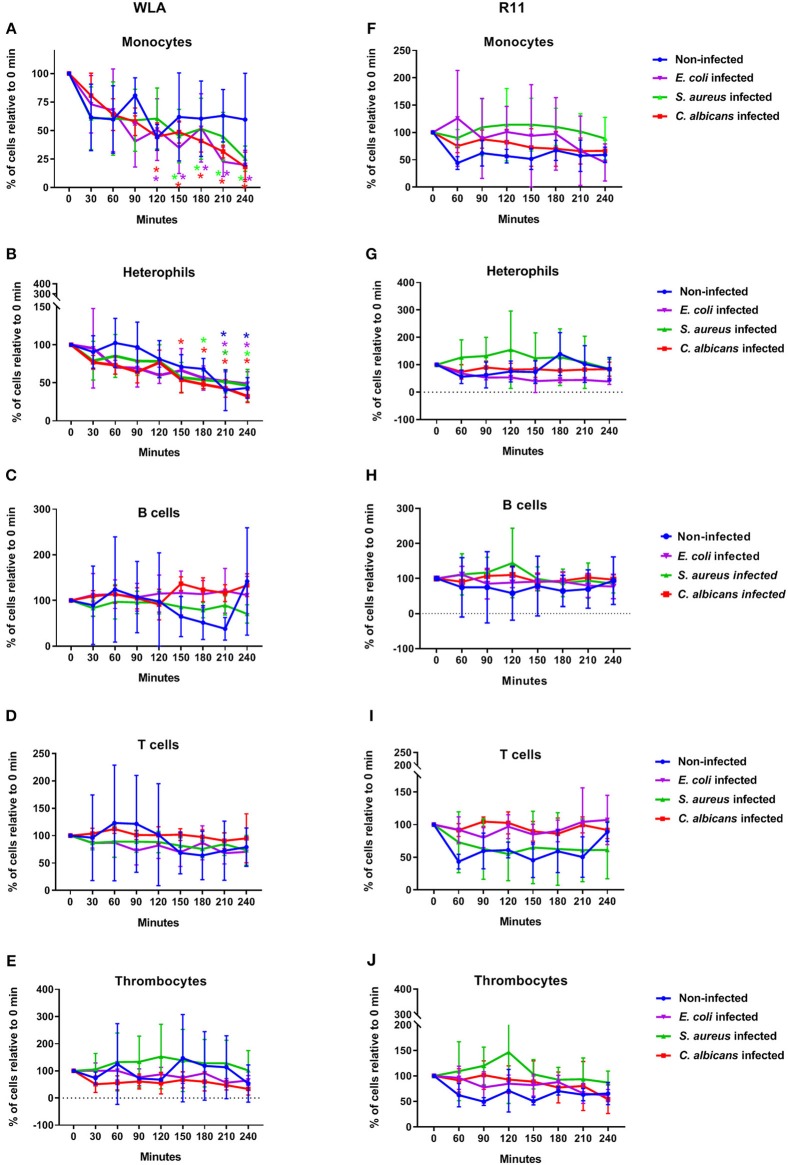
Immune cell numbers in avian whole blood over the course of infection. Avian whole blood from the chicken lines WLA (left) and R11 (right) was infected *ex-vivo* with *C. albicans, S. aureus*, or *E. coli* for 240 min. Absolute numbers were determined for the different immune cell populations using flow cytometry and are depicted in percentage of the numbers at 0 min: Monocytes **(A,F)**, heterophils **(B,G)**, B cells **(C,H)**, T cells **(D,I)**, and thrombocytes **(E,J)**. Data of six independent experiments using blood from different donors is presented as mean and SD. *indicates significant difference compared to 0 min (*p* < 0.05) with the color representing the respective condition: Blue: non-infected blood, purple: *E. coli*, green: *S. aureus*, red: *C. albicans*.

Next, we determined whether infection with *C. albicans, S. aureus*, or *E. coli* affected the number of immune cells. In WLA chickens, a time dependent decrease in monocytes and heterophils was observed during the course of infection (Figures 2A,B). For the *E. coli* infection, the monocyte numbers were significantly lower at 210 and 240 min after infection compared to the non-infected samples (*p* = 0.005 and 0.049, respectively, two-sided unpaired *t*-test). The numbers of other leukocytes and thrombocytes in infected WLA blood remained stable (Figures 2C–E). Infection did not significantly affect leukocyte and thrombocyte numbers in the blood samples of R11 chickens (Figures 2F–J), but a moderate non-significant decrease of monocytes and heterophils in blood infected with *E. coli* was observed (Figures 2F,G). Thus, line-specific and pathogen-specific differences in the viability of monocytes and heterophils following infection were observed.

### Pathogens Are Killed in Avian Blood

**Figure 3:**
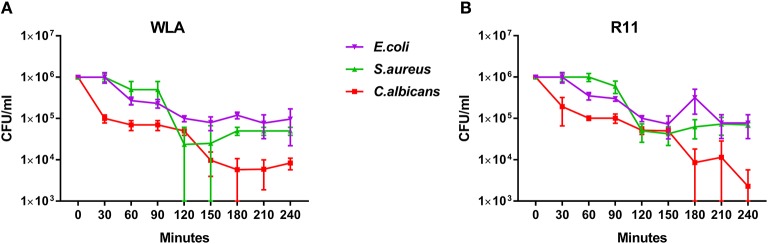
Survival of different pathogens in avian blood. Colony forming units of the different pathogens were determined in the inoculum (0 min) and from samples taken every 30 min to 240 min after infection of whole blood from WLA chickens **(A)** and R11 chickens **(B)**. Data of six independent experiments using blood from different donors is presented as mean and SD.

Next, we determined to which extent the different pathogens survived in avian blood. The number of viable pathogens determined by CFU declined over time in both chicken lines and for all pathogens used (Figures 3A,B). The highest and fastest killing rate was observed for *C. albicans*, which was significantly reduced within the first 30 min and killed more efficiently than both *S. aureus* and *E. coli* during the early phase of infection (Supplementary Table 2). After the initial drop in *C. albicans* CFU 30 min after infection, CFU slowly declined until 120 min (WLA) or 150 min (R11) after infection, followed by a more pronounced decline toward the next time point, suggesting biphasic killing kinetics.

Similar biphasic pathogen survival was observed for *S. aureus*: CFU remained relatively stable until 90 min after infection followed by a steep decline to 120 min, which was more pronounced in WLA blood. Following this decline, CFU remained stable until the end of the experiment in blood from R11 chickens, but increased again in WLA blood, resulting in similar numbers of *S. aureus* in the blood of both chicken lines at the end of the experiment (Figures 3A,B, Supplementary Table 2). In contrast, the number of viable *E. coli* cells showed a steadier decline starting 60 min after infection in both chicken lines. Thus, while all pathogens were killed to a certain extent in avian blood, the killing rates and dynamics differed significantly depending on the pathogen, without differences between the chicken lines.

### Association of Pathogens With the Different Types of Leukocytes Is Pathogen and Chicken Line Dependent

One possible explanation for the different survival rates of the different pathogens in avian blood could be differences in the interaction with leukocytes, which was assessed by flow cytometry.

**Figure 4:**
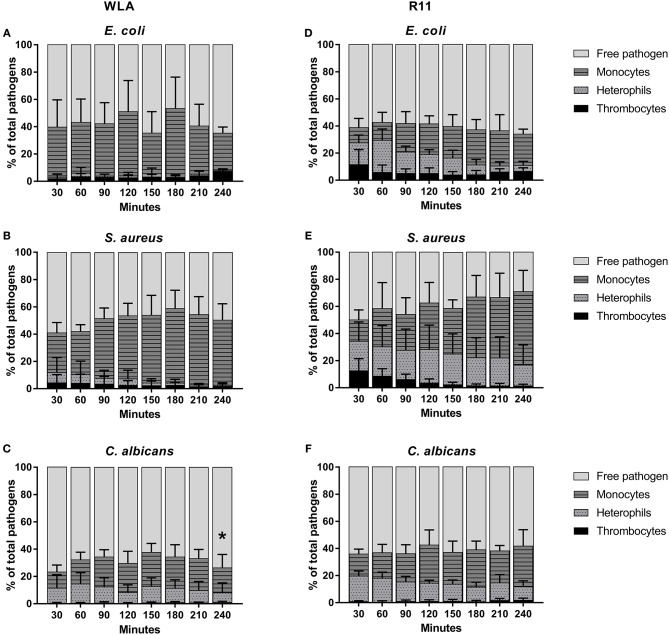
Association of pathogens with host cells in whole blood. Association of *E. coli* **(A,D)**, *S. aureus* **(B,E)** and *C. albicans* **(C,F)** with host cells in whole blood of WLA (left) and R11 chickens (right) was determined by flow cytometry and is presented as percentage of pathogens associated with the host cell type relative to the total pathogen population in blood. Data of six independent experiments using blood from different donors is presented as mean and SD. * indicates significance of monocytes compared to R11 chickens (*p* < 0.05).

Clear differences between WLA and R11 chickens were observed for *E. coli* and *S. aureus*: While both bacterial pathogens were predominantly found in association with monocytes in blood of WLA chickens (Figures 4A,B), a substantial proportion associated with heterophils in the blood of R11 chickens (Figures 4D,E). The relative number of *E. coli* cells detected as being associated with heterophils in R11 blood decreased over time, which could have been caused by either disassociation or killing-mediated loss of the fluorescence signal. In the blood of both WLA and R11 chickens, the overall number of *S. aureus* associated with monocytes increased moderately over time, but remained more stable for *E. coli*. For *C. albicans*, a similar association pattern was observed in both chicken lines: Fungal cells were found to be associated to a slightly higher extent with monocytes than with heterophils (Figures 4C,F). The fraction of fungal cells associated to immune cells was stable after 30 min until the end of the experiment. All pathogens also interacted with thrombocytes, but this interaction was more prominent for the bacterial species than for *C. albicans*. Association with thrombocytes at early time points was more profound in R11 blood. None of the three pathogens was found to be associated with T or B cells in the blood of either of the chicken lines.

Taken together, the data showed that all pathogens associated with monocytes, while the rate of interaction with heterophils was chicken line and pathogen-dependent. Association occurred rapidly and became relatively stable after reaching a certain time point.

### Mathematical Modeling Revealed Relevant Immune Reactions

Based on the measurements conducted in the experimental whole-blood infection assay, we developed different virtual infection models (see Materials and Methods section). We defined different state-based models (SBMs) for fungal infection, involving the switch to hyphal form with rate Ψ, as this morphological transition was observed in blood smears (Supplementary Figure 2). For bacterial infection, bacterial proliferation with rate was included. Furthermore, we defined models that differ in their killing mechanisms of pathogens in extracellular space. We implemented the SEK-SBMs, containing a single extracellular killing mechanism for pathogens, and the MEK-SBMs with multiple extracellular killing mechanisms for pathogens (see Materials and Methods section). Since a decrease in immune cells counts in the non-infected samples was only observed for heterophils in WLA chickens (see Figure 2B), we calibrated the SBM to heterophil kinetics of uninfected samples of WLA chickens and could predict the value of this killing rate of heterophils caused by stress ($\kappa_{stress}^{He}$). Therefore, we set the value of $\kappa_{stress}^{He}=2.6\times 10^{-2}\;s^{-1}$ for infection scenarios with WLA chickens. For infection of samples from R11 chicken we set this rate to zero ($\kappa_{stress}^{He}=0\;s^{-1}$), because the immune cell numbers remain fairly constant over time in the non-infected samples (see Figures 2F,G). A complete list of the resulting transition rate values for all models is provided in Supplementary Tables 3–7.

#### Multiple Extracellular Killing Mechanisms Essential to Resemble Survival of Pathogens

**Figure 5:**
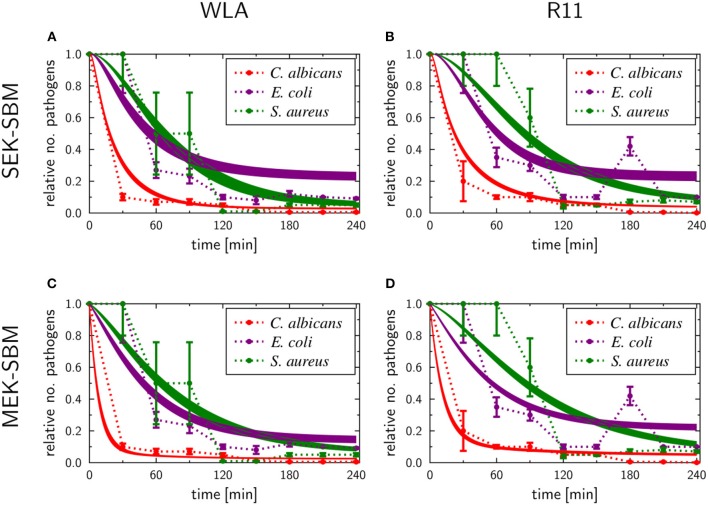
SBM simulations for survival of different pathogens. Time courses of living pathogens resulting from the simulations with the SEK-SBM (single extracellular killing mechanism of pathogens) **(A,B)** and the MEK-SBM (multiple extracellular killing mechanisms of pathogens) **(C,D)**. Solid lines represent SBM simulations that were calibrated to experimentally measured data on pathogen survival (data points that were connected by dashed lines as guide for the eye). The thickness of the solid lines represents the mean ± standard deviation of simulation results observed from 50 simulations for normally distributed transition rates. The models were calibrated to measurements of either *C. albicans* (red lines), *E. coli* (purple lines) or *S. aureus* (green lines) that were injected into samples from WLA chickens (left column) and R11 chickens (right column).

**Figure 6:**
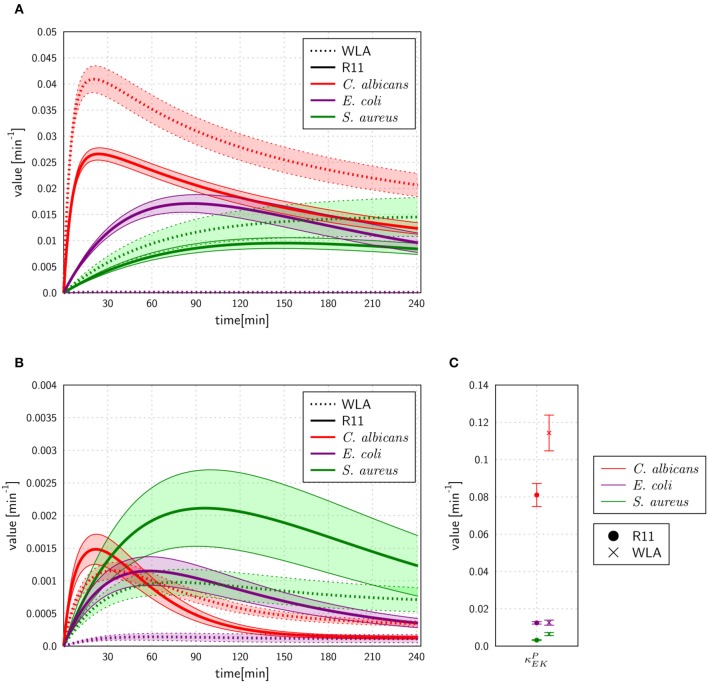
Rates for extracellular killing of pathogens predicted by the SEK-SBM **(A)** and the MEK-SBM **(B,C)**. The time course of extracellular killing caused by antimicrobial peptides that were released upon initial phagocytosis by heterophils [with rate $\kappa_{EK}^{P}\left(t\right)$] is depicted in **(A)** for the SEK-SBM and in **(B)** for the MEK-SBM. The latter SBM additionally contains the mechanism of extracellular killing by peptides that were present immediately upon infection. The predicted values of the respective rate $\kappa_{EM}^{P}$ are shown for the different infection scenarios in **(C)**.

The experimental measurements on survival of pathogens revealed that for all infection scenarios the number of pathogens decreased during the length of the infection assay (see Figures 3, 5). We calibrated the SEK-SBMs and the MEK-SBMs to the experimental measurements and found that simulations of both models were qualitatively in line with the experimental data (see Figure 5). Both SBMs predicted not only a decrease of viable pathogens but also that for both chicken lines *C. albicans* cells were killed faster and to a larger amount than bacterial cells. The values for the extracellular killing rates were also higher for fungal than for bacterial infection, as shown in Figure 6A for SEK-SBM and in Figures 6B, C for MEK-SBM. Note that for the *C. albicans* infection in the MEK-SBMs, the value of $\kappa_{EM}^{P}$ was largest, but not the values of $\kappa_{EM}^{P}\left(t\right)$. Despite these similar qualitative predictions for *C. albicans* infection, data simulated by the SEK-SBMs caused larger least squares error (LSE) in the combined unit of alive pathogens *P*_*A*_ (Supplementary Figures 3A,B) and also a larger total LSE (Supplementary Figure 4A) in comparison to the MEK-SBMs. This is caused by a larger deviation of the simulated data from SEK-SBM to the experimental data from 0 to 60 min after infection (Figures 5A,B) in comparison with the MEK-SBM (Figures 5C,D). Moreover, for this infection scenario, the MEK-SBM showed a smaller *AIC*_*C*_ than the SEK-SBM (Supplementary Figure 4D) indicating that the improvement in terms of the LSE by the MEK-SBM can compensate for the increase in model complexity in comparison to the SEK-SBM. For a direct comparison of simulations of both SBMs we refer to Supplementary Figure 5. Further differences between the simulations by the two SBMs are visible for the number of alive *E. coli* during infection of WLA chicken (Supplementary Figure 5C). Simulations by the SEK-SBM caused a larger *AIC*_*C*_ and larger LSE values for the combined unit *P*_*A*_ (Supplementary Figure 3) and the sum (Supplementary Figure 4), which was mainly caused by increasing deviations to the experimental data starting 120 min after infection (Figure 5A) in comparison to the MEK-SBM (Figure 5C). Even though the values of the extracellular killing rate $\kappa_{EM}^{P}\left(t\right)$ are predicted to be higher in the SEK-SBM than in the MEK-SBM (Supplementary Figure 6), the MEK-SBM simulations showed a more rapid decrease of alive *C. albicans* cells and *E. coli* cells because of the additional extracellular killing rate $\kappa_{EM}^{P}$, which enables pathogen killing immediately upon infection without any temporal shift. Both SBMs predict a similar decrease of alive *S. aureus* cells during infection (Figure 5, Supplementary Figure 5). Of note, none of the SBMs could simulate the biphasic course of *S. aureus* killing that was observed for infection of samples from both chicken lines.

#### Phagocytosis Rates of Immune Cells Are Pathogen-Specific and Differ Quantitatively

**Figure 7:**
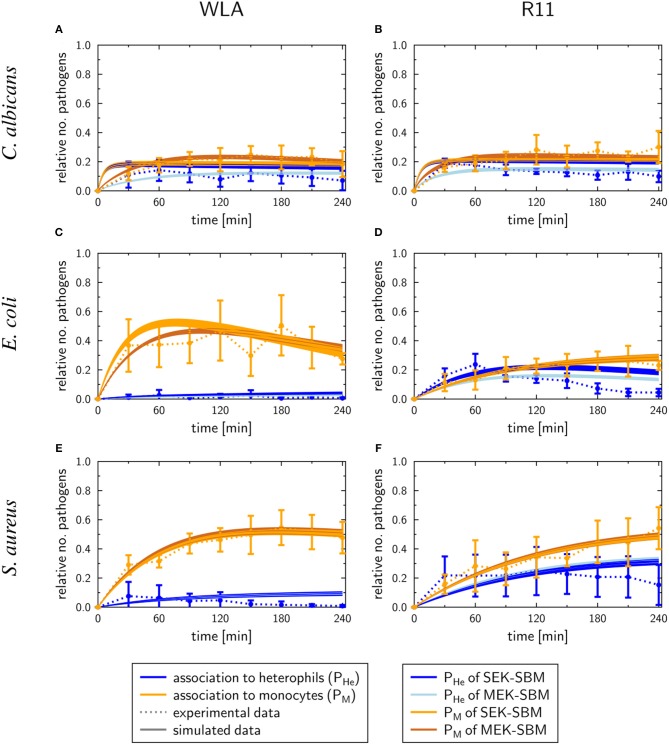
SBM simulations for pathogen association to immune cells. Time courses of the combined units for pathogens in heterophils (*P*_*He*_) and pathogens in monocytes (*P*_*M*_) resulting from the simulations with the SEK-SBM (single extracellular killing mechanism of pathogens) and the MEK-SBM (multiple extracellular killing mechanisms of pathogens). Solid lines represent SBM simulations that were calibrated to experimentally measured data (data points connected by dashed lines as guide for the eye). The thickness of the solid lines represents the mean ± standard deviation of simulation results observed from 50 simulations for normally distributed transition rates. The models were calibrated to measurements of either *C. albicans* **(A,B)**, *E. coli* **(C,D)** or *S. aureus* **(E,F)** that were injected into samples from WLA chickens (left) and R11 chickens (right).

**Figure 8:**
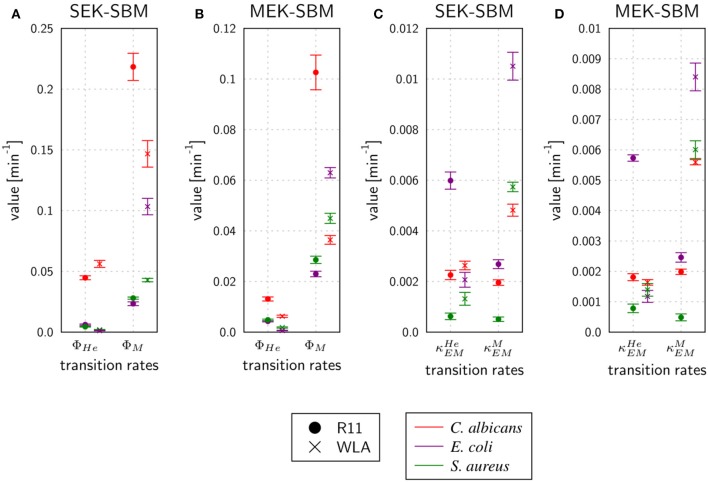
Rates for phagocytosis of pathogens and immune cell killing by extracellular mechanisms predicted by the SEK-SBM **(A,C)** and the MEK-SBM **(B,D)**. Predicted mean values and standard deviations (error bars) of phagocytosis rates of heterophils (Φ_*He*_) and monocytes (Φ_*M*_) **(A,B)** and the rate of heterophil killing ($\kappa_{EM}^{He}$) and monocyte killing ($\kappa_{EM}^{M}$) by extracellular mechanisms that are stimulated in the presence of pathogens **(C,D)**.

As shown in Figure 7, the SEK-SBMs and the MEK-SBMs can be calibrated to the experimental data of pathogen association to immune cells so that the respective simulations are in qualitative agreement with the experimental data. However, we observed quantitative differences for the infection of samples from both chicken lines with *C. albicans*. In comparison to the MEK-SBM, the SEK-SBM simulated larger fractions of fungal cells that were associated to heterophils (Figures 7A,B, Supplementary Figures 7A,B), causing larger deviations from the experimental data as reflected by a larger LSE for the combined unit *P*_*HE*_ (Supplementary Figures 3A,B). The larger fraction of pathogens phagocytosed by heterophils is caused by higher phagocytosis rates in the SEK-SBM in comparison to the MEK-SBM (Figures 8A,B, Supplementary Figures 9A,D). This is also applicable to phagocytosis by heterophils during *E. coli* infection of samples from R11 chicken (Figure 7D, Supplementary Figures 7D, 8E) and phagocytosis by monocytes during *E. coli* infection of samples from WLA chicken (Figure 7C, Supplementary Figure 9, Figures 8A,B, Supplementary Figure 8B). However, both models, the SEK-SBMs and the MEK-SBMs, predicted that not only the fraction of phagocytosed pathogens was larger for monocytes than for heterophils (Figure 7), but also the corresponding functional parameters, i.e., the phagocytosis rates, were larger for monocytes (Φ_*M*_) in comparison to heterophils (Φ_*He*_) for all infection scenarios (Figures 8A,B). The SEK-SBMs predicted that Φ_*M*_ is larger than Φ_*He*_ with at least a factor of Φ_*M*_/Φ_*He*_ = 2.6 for WLA infection with *C. albicans* and up to a factor of Φ_*M*_/Φ_*He*_ = 89.8 for WLA infection with *E. coli* (see Supplementary Table 8 for all other infection scenarios). The MEK-SBMs predicted even larger differences between the phagocytosis rates, with at least Φ_*M*_/Φ_*He*_ = 5.4 for infection with *E. coli* in R11 blood and up to Φ_*M*_/Φ_*He*_ = 101.7 for WLA blood infection with *E. coli*. Furthermore, both models predicted that the phagocytosis rates of heterophils (Φ_*He*_) were higher for bacterial infection of R11 blood compared to WLA (Figures 8A,B, Supplementary Table 9). For fungal infection, the SEK-SBM predicted that Φ_*He*_ is larger in WLA chickens than in R11 chickens (Figure 8A). In contrast, the MEK-SBM predicted the opposite relation of Φ_*He*_ between the chicken-lines.

Taken together, the mathematical models predicted that the experimentally observed chicken-line specific association to heterophils was caused by chicken-line specific phagocytosis rates and not by differences in the immune cell numbers.

#### Immune Cell Killing Mechanism Is Essential to Simulate Immune Response in Avian Whole-Blood Infection

**Figure 9:**
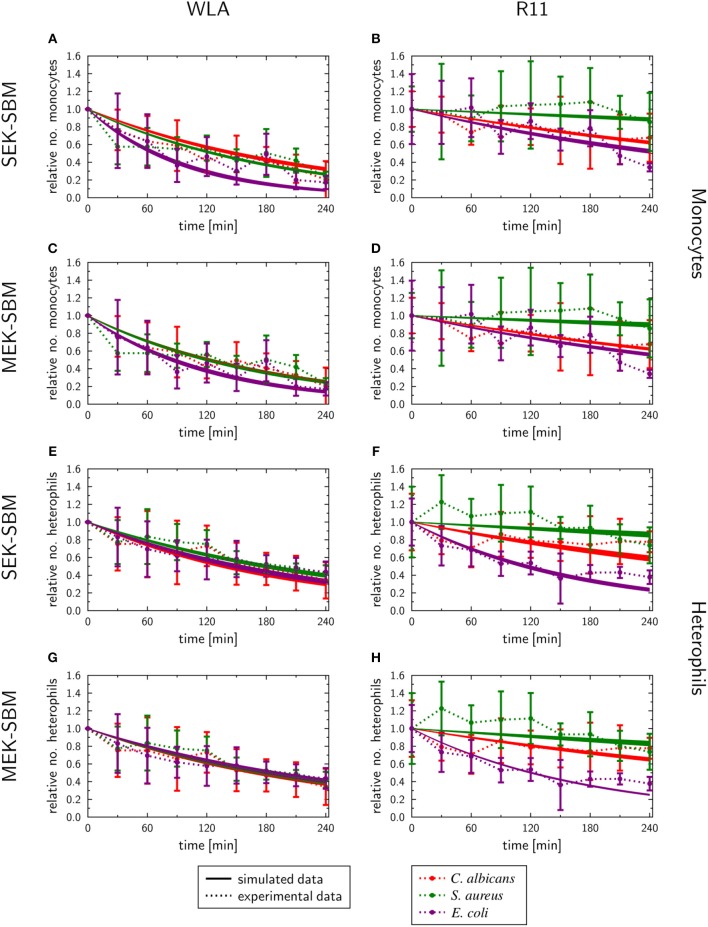
SBM simulations of the immune cell numbers during infection. Time course of the monocytes **(A–D)** and heterophils **(E–H)** predicted units for pathogens in heterophils (*P*_*He*_) and pathogens in monocytes (*P*_*M*_) that were simulated by the SEK-SBM (single extracellular killing mechanism of pathogens) and the MEK-SBM (multiple extracellular killing mechanisms of pathogens). Solid lines represent SBM simulations that were calibrated to experimentally measured data (data points connected by dashed lines as guide for the eye). The thickness of the solid lines represents the mean ± standard deviation of simulation results observed from 50 simulations for normally distributed transition rates. The models were calibrated to measurements of either *C. albicans* cells (red lines), *E. coli* cells (purple lines) or *S. aureus* cells (green lines) that were injected into samples from WLA chickens (left column) and R11 chickens (right column).

As measured using flow cytometry, we observed chicken line-specific and pathogen-specific characteristics in the kinetics of immune cell counts during whole-blood infection (see section “3.1 Impact of infection on leukocyte numbers”). For infection with any of the three pathogens, the number of monocytes and heterophils decreased faster in WLA blood than in samples from R11 chickens (Figure 2, Figure 9, Supplementary Figures 10, 11). The predicted monocyte killing rates ($\kappa_{EM}^{M}$) of both SBMs were higher in infection scenarios with samples from WLA chickens than in samples from R11 chickens (Figures 8C,D, Supplementary Table 10). This relation was not found for the killing rates of heterophils ($\kappa_{EM}^{He}$). Here, higher killing rates were observed in WLA than in R11 blood only for the *S. aureus* infection. Furthermore, we observed that in *E. coli* infected R11 blood the number of both immune cell types decreased faster compared to both *S. aureus* and *C. albicans* cells (Figure 1). As shown in Figure 8 and Supplementary Table 11, these pathogen-specific characteristics are likely due to higher immune cell killing rates during *E. coli* infection.

In order to test whether the mechanism of immune cell killing is essential for avian whole-blood infection, we excluded this mechanism from the fungal and bacterial MEK-SBMs and calibrated the adapted model to the experimental time-series data. However, we observed that in this case the simulations resemble neither the kinetics of heterophil (Supplementary Figure 12) nor monocyte counts (Supplementary Figure 13).

Furthermore, we considered whether the decrease of immune cells can be caused by intracellular pathogens. In case of *C. albicans* cells, we assumed that intracellular hyphae formation can cause immune cell lysis. We adapted the MEK-SBMs by implementing immune cell lysis that is caused by viable, intracellular pathogens and calibrated this model to the experimental measurements. We found that this model does not notably increase the agreement with the experimental data in terms of LSE and moreover showed a larger *AIC*_*C*_ in comparison to the MEK-SBM (Supplementary Figure 14) due to the larger number of model parameters. We also tested whether immune cell lysis only can explain the immune cell kinetics. This was realized by deleting the mechanism of immune cell killing by extracellular factors with rates $\kappa_{EM}^{M}$ and $\kappa_{EM}^{He}$. However, as shown in Supplementary Figures 15, 16, this model does not resemble the immune cell kinetics during bacterial and fungal infection.

#### Expression of Genes Encoding Immune-Related Effectors

**Figure 10:**
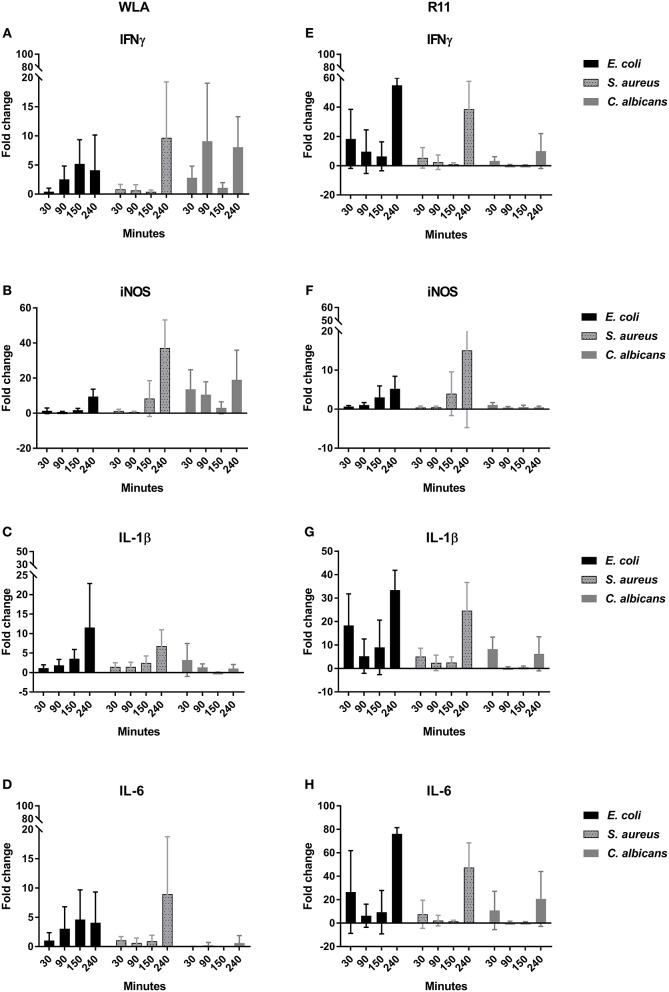
Expression of the genes encoding IFNγ **(A,E)**, iNOS **(B,F)**, IL-1β **(C,G)**, and IL-6 **(D,H)** in infected chicken blood. Left: WLA chickens; right: R11 chickens. Gene expression was normalized to GAPDH and expressed as fold change compared to non-infected samples. The graphs represent the fold change of gene expression in infected avian whole blood relative to non-infected blood samples at the respective time points. Data of six independent experiments using blood from different donors is presented as mean and SD.

To determine whether the association of pathogens with immune cells induced inflammatory responses in whole blood, the transcription of the pro-inflammatory cytokines IFNγ, IL-1β, IL-6, the chemokines IL-8 (CXCLi2), K60 (CXCLi1), and MIP-1β, the effector iNOS, and the central transcription factor LITAF (the avian TNF homolog) was analyzed by quantitative RT-PCR (Figure 10, Supplementary Figure 17). Both chicken lines responded to pathogen challenge with increased gene expression, which was generally more pronounced in the blood of R11 chickens. A notable exception was iNOS, which was upregulated to a lower extent in R11 blood cells. Following *S. aureus* infection, the kinetics of gene induction were also comparable between both cell lines, but differences were observed in blood challenged with *E. coli* or *C. albicans*, respectively: In response to *E. coli*, increased expression of IFNγ, IL-1β, IL-6, IL-8, K60, and MIP1β was observed in R11 blood cells already at early time points, whereas a more gradual increase was observed in WLA blood. Infection with *C. albicans* led to early upregulation of all factors analyzed in WLA blood, with the exception of IL-1β and IL-6, which were not induced by infection (Figure 10). The level of induction was comparable to or higher than those observed post infection with *E. coli* or *S. aureus*. In contrast, IL-1β and IL-6 were induced by *C. albicans* in blood cells of R11 chickens, but the induction of these and all other genes analyzed was less pronounced in response to *C. albicans* compared to both bacterial species. Thus, while both chicken lines responded to all pathogens by increased expression of genes associated with immune reactions, both pathogen- and chicken-line dependent differences were observed.

## Discussion

The aim of this study was to better understand the interaction of model pathogens with avian blood as an important step in the pathogenesis of disseminated infections and during bacteremia. Therefore we employed an *ex vivo* whole-blood infection assay in combination with mathematical infection modeling. The advantage of the experimental whole-blood assay is that it enables identification of the immune cells that interact with a pathogen in a complex setting allowing for cross talk of immune cells. Furthermore, the absence of isolation and purification steps prevents accidental pre-activation of immune cells that could occur in the use of primary cells isolated from blood (52). Our set up was similar to a recently published approach for measuring phagocytic activity of chicken leukocytes (53), with the differences that we (i) discriminated between various immune cell populations, and (ii) performed a time course analysis. It should be noted that neither our method nor the approach by Nagahizadeh et al. (53) can clearly distinguish between attachment of pathogens to and phagocytosis by immune cells. We therefore refer to the biological interactions observed as association rather than phagocytosis. However, it has been shown that association of *C. albicans* with innate immune cells in human blood usually indicates phagocytosis (17), and it appears likely that this is also the case in chicken blood not only for *C. albicans* but also for the bacterial pathogens. In this context, it should also be noted that the overall association of microbes with immune cells in our model appeared to be relatively stable over time. However, this does not indicate stable interactions on a cell-to-cell basis, as (i) degradation of the fluorescence signal in killed pathogens would lead to a loss of association of the corresponding immune cell in flow cytometry analysis, (ii) microbes might escape from immune cells, and (iii) free microbes might be taken up by other immune cells.

These possibilities, and the assumption that association is indicative of or leading to phagocytosis, were incorporated into the mathematical model. By mapping the complex biological system of *ex vivo* whole-blood infection into a mechanistic mathematical model, we could not only quantify functional characteristics of the immune response but also identify novel immune mechanisms. Since the knowledge concerning immune mechanisms in avian blood is limited, we started with our established human virtual infection model (17, 28) and stepwise added known as well as potential immune mechanisms. By calibrating these models to experimental measurements and subsequently scoring the models by their agreement with experimental data, using the least squares error (LSE) and the Akaike information criteria confirmed that the immune reactions included in the model were justified and necessary to be able to model the experimental data.

A possible technical concern of the *ex-vivo* whole-blood infection assay is the stability of this model system over time. As a decline in absolute cell numbers was only observed for heterophils, and to a lesser extent monocytes, in WLA chickens over the observation period of 240 min, we can assume that this system is reasonably stable within this time frame, similar to the human *ex vivo* whole-blood model previously described (17). We however accounted for the heterophil decrease by implementing the mechanism of heterophil death caused by stress factors of the experimental setting into the mathematical models. By calibrating the model to heterophil kinetics of non-infected WLA samples we could quantify the corresponding reaction rate and distinguish this rate from immune cell killing caused by infection. Upon infection, a decrease in cell numbers was observed for monocytes and heterophils from WLA chickens, while the immune cell decrease was less pronounced in R11 chickens, except for infection by *E. coli*. Since the virtual infection models differentiated between immune cell killing caused by stress and caused by infection, we could quantify the relative contribution of each pathogen to immune cell killing and the differences between the immune cell types and the chicken lines. We found that in WLA blood, the killing rate of monocytes is higher than that of heterophils; also, more monocytes are killed in WLA than in R11 blood. Moreover, in R11 blood the immune cell killing rate is highest for an *E. coli* infection. Both bacterial pathogens tested also displayed a significantly more pronounced interaction with monocytes in WLA blood compared to R11. Thus, increased interaction with monocytes coincided with a stronger decrease in monocyte numbers, suggesting killing of monocytes by *E. coli* and *S. aureus*. This explanation would contrast results from *in vitro* experiments in which *E. coli* did not lead to detectable chicken macrophage killing within the first 4 h (54). Similarly, the viability of mammalian macrophages is not substantially impaired by infection with *S. aureus* within the first 4 h, even though killing occurs at later time points (55). To our knowledge the fate of avian macrophages challenged with *S. aureus* has not been investigated so far, but assuming that interactions would be similar to those reported for mammalian macrophages, our data could indicate significant differences in the outcome of bacteria-macrophage interactions *in vitro* compared to the *ex vivo* whole-blood model. This could be due to differences between circulating monocytes and the macrophage cell line used for the *in vitro* studies, the bacterial strain used, or immune cell responses might be influenced by the more complex environment in whole blood compared to tissue culture.

However, by adding and removing potential reactions within the mathematical model, we found that immune cell killing is likely not exclusively caused by viable, intracellular pathogens that perform lysis. In addition, immune cell killing caused by extracellular factors that originate from pathogens independent of their viability, was essential to calibrate the model to the experimental data. While we deemed it to be beyond the scope of this study to test these hypotheses experimentally, it highlights how bioinformatical modeling can generate novel hypotheses from complex experimental data that could be tested in future studies.

An unexpected observation was the clear drop of *S. aureus* CFU numbers from 90 to 120 min after infection in both chicken lines. This could have been mediated by intracellular killing of bacteria by immune cells, possibly monocytes, which showed higher association to *S. aureus* in the blood of WLA chickens, which correlates also with the more pronounced reduction in the bacterial CFU counts at this time point. After this reduction, the CFU counts, however, remained stable (R11) or even increased (WLA). Although macrophages can kill *S. aureus, in vitro* experiments using mammalian cells demonstrated that a subpopulation is able to survive in macrophages, before it escapes and replicates extracellularly (55). A similar mechanism would explain the observed kinetics of *S. aureus* CFU in avian blood. So far, the mathematical models could not simulate the biphasic kinetics of viable *S. aureus* cells in avian whole-blood, because the killing and proliferation mechanisms were implemented as reactions with rates that are constant in time. In future studies, these mechanisms could be characterized by time-dependent rates. However, one should keep in mind that this would imply an increase in model complexity. Furthermore, the Next-Reaction simulation algorithm (56), an improved implementation of the original algorithm by Gillespie (57, 58), must be applied to simulate the model dynamics, since the Random Selection method does not accurately simulate systems with time-dependent rates (51).

In comparison to bacterial killing, we found that fungal cells were killed faster and to a larger extent than bacterial cells in both chicken lines. Even taking into account that bacterial cells can proliferate during infection, the predicted killing rates were lower compared to those for fungal infections. Furthermore, we found that multiple extracellular killing mechanisms of pathogens were necessary to calibrate the model to the experimentally measured numbers of viable pathogens. Only the MEK-SBM with multiple extracellular killing mechanisms could accurately simulate the kinetics of alive *E. coli* cells in R11 chicken and alive *C. albicans* cells in R11 and WLA chicken, as also justified by the smallest LSE and the best information criterion *AIC*_*C*_ for these infection scenarios. A likely biological explanation is the release of antimicrobial peptides by activated host cells (59).

As similar/identical characteristics among all infection scenarios, we observed that the degree of pathogen association and the phagocytosis rate is higher for monocytes in comparison to heterophils. This observation clearly reveals differences to the immune responses observed in human whole blood, where monocytes show less association to pathogens and lower phagocytosis rates in comparison to neutrophils (17). However, we also observed chicken-line specific heterophil association and phagocytosis for bacterial infection. Infection with either of the two bacterial species induced a stronger response by heterophils in R11 blood in comparison to WLA blood. Both monocytes/macrophages and heterophils are recruited during bacterial infections *in vivo* and are thought to contribute to pathogen clearance (4). Our results would thus warrant future comparative analyses addressing both the relative contribution of either type of innate immune cells to pathogen killing and the potential differences depending on the genetic background. Future studies could also address whether different types of immune cells respond to a different degree to bacterial vs. fungal pathogens as we observed a higher degree of association of bacterial pathogens with monocytes than heterophils.

Heterophil interaction might be essential for reducing fungal burden, as *C. albicans* differs from both *S. aureus* and *E. coli* in its *in vitro* interaction with macrophages: *C. albicans* kills 20-50% of macrophages within the first hours of interaction *in vitro* (60, 61). This early macrophage killing by *C. albicans* is mediated by pyroptosis, a type of programmed cell death. Whether this process can also occur in avian macrophages is unclear (62), but it would explain the reduction of monocytes upon *Candida* infection in WLA blood. The declining number of monocytes however does not exclude contribution of these cells to fungal killing in our model; rapid phagocytosis by monocytes/macrophages (63) and macrophage efficacy against *Candida* species have been demonstrated previously (64), making it likely that avian monocytes/macrophages contribute to fungal clearance.

Due to the limited capacity of macrophages to control *C. albicans*, neutrophils are considered to be the main effector cells during candidiasis in mammals (17, 65, 66). They are also by far the dominating cell type associated with *C. albicans* in human blood, where monocytes comprise only a minor fraction of the cells interacting with the fungus (17). Avian heterophils can rapidly phagocytose and inactivate *C. albicans* (67, 68), and, additionally, antimicrobial peptides of heterophils have been shown to be effective against *C. albicans* (69). Chicken serum alone, in contrast, does not inhibit *Candida* (68). Thus, release of antimicrobial peptides following degranulation of heterophils could explain the significant killing of *C. albicans* cells. It should also be noted, that all microbes used in this study were cultured in standard media under standard conditions, and that the pathogens have to adapt to the altered environment when inoculated into the blood. Clinical blood stream infections with these pathogens in contrast usually originate from mucosal sites, such as the gut or the respiratory tract. Adaptation to these niches might better prepare the microorganisms for the interactions with immune cells once they enter the blood stream (70–73).

The nucleated thrombocytes of avian species also contribute to the overall immune response in blood by phagocytosis of pathogens and upregulation of proinflammatory cytokines (4, 74). We did, however, only observe low association rates of pathogens with thrombocytes in our model, making it unlikely that these cells make a significant direct contribution to microbial clearance. Nonetheless, thrombocytes might be important for the overall host response by influencing other immune cells, for example, by the release of stimulating cytokines. Expression of cytokine genes and genes encoding other immune-related factors was increased in whole blood following infection, consistent with the previously reported induction of proinflammatory cytokines in human whole blood infected with *C. albicans* (17). As our data was based on mRNA analysis of whole blood, it however remains unknown which cells in the model are responsible for the observed upregulation. Without cell type-based analysis, it is furthermore not possible to determine the reason for the observed differences between the chicken lines; these might be due to the differences in association of pathogens with the different types of immune cells, leading to differences in the number of cells of a given subset being activated by physical contact to microbes. Also, heterophils and monocytes can be expected to differ in their transcriptional responses both qualitatively and quantitatively, so that differences in the extent of association could affect the overall transcriptional response. It is however also conceivable that distinct types of immune cells in the two chicken lines used in this study differ in their response to pathogens, as has previously been demonstrated for other chicken lines (75–77). This possibility would have to be tested using isolated subsets of immune cells.

In summary, we describe here an *ex vivo* avian whole-blood infection assay analyzed by flow cytometry in combination with biomathematical modeling. Our results provide first insights into the interaction of three model pathogens with different immune cell populations in chicken blood, demonstrating differences depending not only on the pathogen but also on the chicken line. Furthermore, microbial clearance rates differed between the pathogens. The application of mechanistic virtual infection modeling predicted essential and novel immune mechanisms. It should be noted that our study focused only on a few factors (physical interaction with immune cells and expression of selected cytokines) that affect the outcome of host-pathogen interaction in this complex model. The contribution of important immune effector mechanisms such as complement or the release of antimicrobial peptides (e.g., lysozyme) were not addressed. Analyzing complement activation and antimicrobial peptides will likely provide important further insights in the activation and relevance of these host defense mechanisms. Furthermore, analysis of the global transcriptional changes, for example by using sequencing approaches, would provide a more comprehensive overview on the reaction of cells in whole blood during infection. To elucidate the functional importance of the associations observed as well as the underlying molecular mechanisms, it would be helpful to selectively deplete distinct types of immune cells and/or to functionally analyze immune cells isolated from naïve and infected blood.

## Data Availability Statement

All datasets generated for this study are included in the article/Supplementary Material.

## Ethics Statement

The protocol was approved by the Committee on the Ethics of Animal Experiments and the Protection of Animals of the State of Thuringia, Germany (permit number 04-001-14).

## Author Contributions

IJ, AB, and MF conceived the study. SS, TL, MP, and CB performed the experiments. SS, TL, MP, IJ, AB, and MF analyzed the data. IJ and TL drafted the manuscript. SS, TL, MP, AB, CB, MF, and IJ revised and approved the manuscript.

### Conflict of Interest

The authors declare that the research was conducted in the absence of any commercial or financial relationships that could be construed as a potential conflict of interest.

## Supplementary Material

Datasheet 1 Tables.

Datasheet 2 Supplementary methods and figures.
