# Supplementary material for: Dynamic Interplay of Host and Pathogens in an Avian Whole-Blood Model
Source: Front Immunol. 2020 Mar 31;11:500. doi: 10.3389/fimmu.2020.00500 (PMC7136455; doi:10.3389/fimmu.2020.00500)
Supplement: Datasheet 1 — Tables. [file Data_Sheet_1.pdf]

frontiers

Supplementary Tables

Supplementary Table 1: Absolute numbers of immune cell in non-infected chicken blood. Whole blood of WLA and R11 chickens was collected and immune cells were measured every 30 to 60 min until 240 min post collection by flow cytometry. Absolute numbers of viable monocytes, granulocytes, thrombocytes, T and B cells per µl of blood are presented as mean and SD of the data derived from six independent experiments using different donors.

WLA chickens									
	0 min	30 min	60 min	90 min	120 min	150 min	180 min	210 min	240 min
Thrombocytes	$1.79\times10^4 \pm 1.12E\times10^4$	$1.82\times10^4 \pm 3.60\times10^3$	$1.23\times10^4 \pm 2.46\times10^3$	$2.12\times10^4 \pm 5.59\times10^3$	$1.78\times10^4 \pm 3.24\times10^3$	$1.20\times10^4 \pm 2.86\times10^4$	$1.00\times10^4 \pm 2.53\times10^3$	$1.05\times10^4 \pm 4.09\times10^3$	$5.11\times10^4 \pm 3.91\times10^4$
T cells	$1.20\times10^4 \pm 5.63\times10^3$	$8.48\times10^3 \pm 2.64\times10^3$	$1.04\times10^4 \pm 3.75\times10^3$	$1.09\times10^4 \pm 3.03\times10^3$	$8.88\times10^3 \pm 4.02\times10^3$	$7.63\times10^3 \pm 1.20\times10^3$	$6.96\times10^3 \pm 1.83\times10^3$	$7.35\times10^3 \pm 2.40\times10^3$	$8.16\times10^3 \pm 2.58\times10^3$
B cells	$2.84\times10^3 \pm 1.62\times10^3$	$1.86\times10^3 \pm 9.76\times10^2$	$2.29\times10^3 \pm 9.33\times10^2$	$2.22\times10^3 \pm 7.24\times10^2$	$1.98\times10^3 \pm 1.29\times10^3$	$1.83\times10^3 \pm 6.50\times10^2$	$1.53\times10^3 \pm 1.20\times10^3$	$1.70\times10^3 \pm 6.67\times10^2$	$2.90\times10^3 \pm 1.21\times10^3$
Monocytes	$1.59\times10^3 \pm 6.85\times10^2$	$8.21\times10^2 \pm 1.38\times10^2$	$8.34\times10^2 \pm 2.59\times10^2$	$1.40\times10^3 \pm 3.42\times10^2$	$9.51\times10^2 \pm 5.79\times10^2$	$8.48\times10^2 \pm 3.18\times10^2$	$8.15\times10^2 \pm 3.27\times10^2$	$1.11\times10^3 \pm 3.23\times10^2$	$7.75\times10^2 \pm 3.32\times10^2$
Heterophils	$4.31\times10^3 \pm 6.83\times10^2$	$3.80\times10^3 \pm 3.79\times10^2$	$4.26\times10^3 \pm 6.67\times10^2$	$4.00\times10^3 \pm 6.14\times10^2$	$3.39\times10^3 \pm 5.11\times10^2$	$2.98\times10^3 \pm 3.90\times10^2$	$2.87\times10^3 \pm 2.72\times10^2$	$1.64\times10^3 \pm 9.19\times10^2$	$1.83\times10^3 \pm 5.57\times10^2$
R11 chickens									
	0 min		60 min	90 min	120 min	150 min	180 min	210 min	240 min
Thrombocytes	$3.59\times10^4 \pm 1.67\times10^4$		$2.19\times10^4 \pm 3.75\times10^3$	$2.09\times10^4 \pm 4.09\times10^3$	$2.31\times10^4 \pm 7.26\times10^3$	$2.12\times10^4 \pm 3.83\times10^3$	$2.92\times10^4 \pm 1.73\times10^3$	$2.61\times10^4 \pm 2.87\times10^3$	$2.66\times10^4 \pm 4.94\times10^3$
T cells	$9.69\times10^3 \pm 1.97\times10^3$		$4.24\times10^3 \pm 1.69\times10^3$	$5.42\times10^3 \pm 8.20\times10^2$	$6.14\times10^3 \pm 1.88\times10^3$	$4.78\times10^3 \pm 2.80\times10^3$	$6.59\times10^3 \pm 7.08\times10^2$	$5.75\times10^3 \pm 4.54\times10^2$	$8.52\times10^3 \pm 1.44\times10^3$
B cells	$1.72\times10^3 \pm 7.62\times10^2$		$1.63\times10^3 \pm 1.44\times10^3$	$1.50\times10^3 \pm 1.48\times10^3$	$1.30\times10^3 \pm 1.17\times10^3$	$1.64\times10^3 \pm 1.45\times10^3$	$1.48\times10^3 \pm 5.02\times10^2$	$1.54\times10^3 \pm 6.29\times10^2$	$1.95\times10^3 \pm 8.15\times10^2$
Monocytes	$1.96\times10^3 \pm 7.26\times10^2$		$8.13\times10^2 \pm 1.43\times10^2$	$9.00\times10^2 \pm 3.41\times10^2$	$8.64\times10^2 \pm 1.58\times10^2$	$8.42\times10^2 \pm 1.88\times10^2$	$1.09\times10^3 \pm 2.02\times10^2$	$9.36\times10^2 \pm 3.18\times10^2$	$9.96\times10^2 \pm 2.86\times10^2$
Heterophils	$5.99\times10^3 \pm 2.54\times10^3$		$3.38\times10^3 \pm 5.61\times10^2$	$3.64\times10^3 \pm 1.95\times10^3$	$4.37\times10^3 \pm 1.16\times10^3$	$4.25\times10^3 \pm 1.00\times10^3$	$7.84\times10^3 \pm 2.20\times10^3$	$5.92\times10^3 \pm 2.12\times10^3$	$4.75\times10^3 \pm 1.05\times10^3$

Supplementary Table 2: CFU counts of the different pathogens in whole blood of WLA and R11 chickens. Colony forming units of the different pathogens were determined in samples collected every 30 min up to 240 min after infection. Data of six independent experiments using blood from independent donors is presented as mean and SD. Statistically significant ($p<0.05$) differences determined by 2-way ANOVA and Tukey's multiple comparison test are indicated as follows: * significant difference to the initial infection dose; # significant difference to the previous time point. Grey background indicates significant difference of *C. albicans* CFU compared to both *S. aureus* and *E. coli* CFU.

**WLA chickens**									
	Initial infection dose	30 min	60 min	90 min	120 min	150 min	180 min	210 min	240 min
C. albicans		1.00×10^{5} ± 2.19×10^{4} *	7.00×10^{4} ± 1.90×10^{4} *	7.00×10^{4} ± 1.90×10^{4} *	5.00×10^{4} ± 1.10×10^{4} *	9.72×10^{3} ± 5.77×10^{3} *	5.77×10^{3} ± 4.92×10^{3} *	5.92×10^{3} ± 4.03×10^{3} *	8.33×10^{3} ± 2.58×10^{3} *
S. aureus	1.00×10^{6}	1.00×10^{6} ± 2.19×10^{5}	5.00×10^{5} ± 2.83×10^{5} *#	5.00×10^{5} ± 2.83×10^{5} *	2.35×10^{4} ± 3.76×10^{4} *#	2.50×10^{4} ± 4.18×10^{4} *	5.00×10^{4} ± 1.10×10^{4} *	5.00×10^{4} ± 1.10×10^{4} *	5.00×10^{4} ± 1.10×10^{4} *
E. coli		1.00×10^{6} ± 2.83×10^{5}	2.70×10^{5} ± 5.72×10^{4} *#	2.33×105 ± 5.38×10^{4} *	1.00×10^{5} ± 1.63×10^{4} *	8.00×10^{4} ± 2.94×10^{4} *	1.20×10^{5} ± 2.16×10^{4} *	7.75×10^{4} ± 4.50×10^{4} *	9.63×10^{4} ± 7.42×10^{4} *

**R11 chickens**									
	Initial Infection dose	30 min	60 min	90 min	120 min	150 min	180 min	210 min	240 min
C. albicans		1.93×10^{5} ± 1.27×10^{5} *	1.01×10^{5} ± 1.21×10^{4} *	1.01×10^{5} ± 2.54×10^{4} *	5.14×10^{4} ± 1.07×10^{4} *	5.00×10^{4} ± 8.16×10^{3} *	8.57×10^{3} ± 9.50×10^{3} *	1.14×10^{4} ± 1.70×10^{4} *	2.29×10^{3} ± 3.40×10^{3} *
S. aureus	1.00×10^{6}	1.00×10^{6} ± 2.19×10^{5}	1.00×10^{6} ± 2.19×10^{5}	6.00×10^{5} ± 2.00×10^{5} *#	5.00×10^{4} ± 2.37×10^{4} *#	4.25×10^{4} ± 2.04×10^{4} *	6.25×10^{4} ± 3.06×10^{4} *	7.25×10^{4} ± 3.34×10^{4} *	7.00×10^{4} ± 8.94×10^{3} *
E. coli		1.00×10^{6} ± 2.83×10^{5}	3.50×10^{5} ± 7.07×10^{4} *#	3.00×10^{5} ± 4.08×10^{4} *	1.00×10^{5} ± 1.63×10^{4} *	7.30×10^{4} ± 4.09×10^{4} *	3.17×10^{5} ± 1.90×10^{5} *	7.75×10^{4} ± 4.50×10^{4} *	7.75×10^{4} ± 4.50×10^{4} *

Supplementary Table 3: The estimated transition rate values of the SEK-SBM (single extracellular killing mechanism of pathogens) for infection of WLA and R11 blood, respectively, with either *C. albicans*, *E. coli*, or *S. aureus*. The transition rates are given by the phagocytosis rates of heterophils (ϕ_{He}) and of monocytes (ϕ_M), the immune-evasion rate ρ, the rates for pathogen killing in monocytes (κ_M), in heterophils (κ_{He}) and in the extracellular space by the parameters $\bar{\kappa}_{EK}$ and γ which, combined, give the extracellular killing rate $\kappa_{EK}^{P}(t)$. Furthermore, pathogens can be killed in extracellular space by temporally constant mechanisms with rate κ_{EK}^{P}. The rates for killing of heterophils and monocytes are given by $\kappa_{stress}^{He}$and κ_{stress}^{M} for killing by stress, by κ_{lysis}^{He} and κ_{lysis}^{M} for killing by pathogen-induced lysis as well as by κ_{EM}^{He} and $\kappa_{EM}^{M}$for killing by extracellular mechanisms that were induced by pathogens. The fungal SBM additionally contains the rate Ψ for the switch from the yeast form to hyphal form, whereas the bacterial SBM contain the rate o for proliferation of bacterial cells. The mean values and standard deviations (SD) of the transition rates were predicted by the global fitting procedure Metropolis Monte Carlo explained in the Methods section. The coefficient of variation (CV) refers to the ratio of the SD to the mean.

	mean $\times 10^{-2}$ $[min^{-1}]$$\pm$ SD $\times 10^{-2}$ $[min^{-1}]$ (CV [%])					
SEK-SBM	WLA chicken			R11 chicken		
Transition rate	*C. albicans*	*E. coli*	*S. aureus*	*C. albicans*	*E. coli*	*S. aureus*
Φ_{He}	5.61 ± 0.288 (5.13)	0.12 ± 0.015 (13.09)	0.19 ± 0.024 (12.39)	4.47 ± 0.163 (3.64)	0.62 ± 0.052 (8.37)	0.44 ± 0.030 (6.64)
Φ_M	14.67 ± 1.092 (7.44)	10.33 ± 0.676 (6.54)	4.28 ± 0.141 (3.29)	21.83 ± 1.117 (5.12)	2.34 ± 0.152 (6.51)	2.80 ± 0.086 (3.09)
κ_M	7.67 ± 0.583 (7.60)	17.38 ± 3.469 (19.96)	8.96 ± 0.776 (8.66)	9.90 ± 1.167 (11.79)	10.36 ± 1.006 (9.71)	5.79 ± 0.992 (17.15)
κ_{He}	38.78 ± 2.932 (7.56)	16.74 ± 4.538 (27.11)	10.90 ± 2.696 (24.73)	36.63 ± 5.006 (13.67)	18.33 ± 2.195 (11.98)	6.11 ± 1.198 (19.61)
ρ	0.65 ± 0.077 (11.80)	0.50 ± 0.064 (12.84)	0.08 ± 0.014 (18.72)	0.62 ± 0.042 (6.77)	0.50 ± 0.062 (12.38)	0.08 ± 0.015 (17.67
γ	1.01 ± 0.060 (5.93)	4.90 ± 0.791 (16.14)	0.61 ± 0.108 (17.87)	0.69 ± 0.046 (6.73)	1.28 ± 0.200 (15.65)	0.56 ± 0.062 (11.08
$\overline{\kappa_{EK}}$	97.56 ± 1.185 (1.21)	14.05 ± 2.461 (17.51)	76.46 ± 10.376 (13.57)	97.49 ± 1.569 (1.61)	96.49 ± 1.219 (1.26)	26.61 ± 2.250 (8.46)
κ_{lysis}^{He}	0.00 ± 0.000 (0.00)	0.00 ± 0.000 (0.00)	0.00 ± 0.000 (0.00)	0.00 ± 0.000 (0.00)	0.00 ± 0.000 (0.00)	0.00 ± 0.000 (0.00)
κ_{stres}^{He}	0.26 ± 0.000 (0.00)	0.26 ± 0.000 (0.00)	0.26 ± 0.000 (0.00)	0.00 ± 0.000 (0.00)	0.00 ± 0.000 (0.00)	0.00 ± 0.000 (0.00)
κ_{lysis}^{M}	0.00 ± 0.000 (0.00)	0.00 ± 0.000 (0.00)	0.00 ± 0.000 (0.00)	0.00 ± 0.000 (0.00)	0.00 ± 0.000 (0.00)	0.00 ± 0.000 (0.00)
κ_{stress}^{M}	0.00 ± 0.000 (0.00)	0.00 ± 0.000 (0.00)	0.00 ± 0.000 (0.00)	0.00 ± 0.000 (0.00)	0.00 ± 0.000 (0.00)	0.00 ± 0.000 (0.00)
Ψ	12.53 ± 0.687 (5.48)	0.00 ± 0.000 (0.00)	0.00 ± 0.000 (0.00)	0.00 ± 0.000 (0.00)	0.00 ± 0.000 (0.00)	0.00 ± 0.000 (0.00)
o	0.00 ± 0.000 (0.00)	0.12 ± 0.020 (16.86)	0.07 ± 0.009 (11.90)	9.59 ± 0.441 (4.60)	0.17 ± 0.018 (10.18)	0.04 ± 0.007 (16.84)
κ_{EK}^{P}	0.00 ± 0.000 (0.00)	0.00 ± 0.000 (0.00)	0.00 ± 0.000 (0.00)	0.00 ± 0.000 (0.00)	0.00 ± 0.000 (0.00)	0.00 ± 0.000 (0.00)
κ_{EM}^{He}	0.26 ± 0.017 (6.48)	0.21 ± 0.029 (14.22)	0.13 ± 0.025 (19.27)	0.23 ± 0.018 (8.16)	0.60 ± 0.034 (5.67)	0.06 ± 0.013 (20.70)
κ_{EM}^{M}	0.48 ± 0.024 (4.94)	1.05 ± 0.055 (5.24)	0.57 ± 0.019 (3.26)	0.20 ± 0.011 (5.60	0.27 ± 0.018 (6.57)	0.05 ± 0.009 (17.35)

Supplementary Table 4: The estimated transition rate values of the MEK-SBM (multiple extracellular killing mechanisms of pathogens) for infection of WLA and R11 blood, respectively, with either *C. albicans*, *E. coli*, or *S. aureus*. The definition of each transition rate is given in Table 1 and in the caption of Table S5. The mean values and standard deviations (SD) of the transition rates were predicted by the global fitting procedure Metropolis Monte Carlo explained in the Methods section. The coefficient of variation (CV) refers to the ratio of the SD to the mean.

	mean × 10^{-2} $[min^{-1}]$± SD × 10^{-2} $[min^{-1}]$ (CV [%])					
**MEK-SBM**	WLA chicken			R11 chicken		
Transition rate	*C. albicans*	*E. coli*	*S. aureus*	*C. albicans*	*E. coli*	*S. aureus*
Φ_{He}	0.62 ± 0.44 (7.09)	0.06 ± 0.11 (17.43)	0.18 ± 0.024 (12.96)	1.31 ± 0.080 (6.11)	0.43 ± 0.024 (5.62)	0.48 ± 0.029 (6.01)
Φ_M	3.64 ± 1.72 (4.73)	6.30 ± 2.05 (3.25)	4.50 ± 0.200 (4.46)	10.26 ± 0.685 (6.68)	2.30 ± 0.110 (4.78)	2.86 ± 0.145 (5.06)
κ_M	3.06 ± 4.16 (13.58)	7.29 ± 6.64 (9.11)	7.53 ± 1.121 (14.89)	1.93 ± 0.260 (13.51)	6.41 ± 0.729 (11.37)	6.12 ± 0.678 (11.09)
κ_{He}	17.60 ± 18.51 (10.52)	8.89 ± 26.04 (29.29)	6.12 ± 1.259 (20.57)	16.20 ± 2.205 (13.62)	7.84 ± 0.858 (10.95)	5.02 ± 0.925 (18.43)
ρ	0.50 ± 0.45 (8.96)	0.37 ± 0.62 (16.64)	0.08 ± 0.009 (10.94)	0.92 ± 0.081 (8.81)	0.51 ± 0.059 (11.56)	0.08 ± 0.018 (23.32)
γ	4.44 ± 2.74 (6.18)	2.99 ± 9.79 (32.75)	2.28 ± 0.450 (19.74)	3.98 ± 0.365 (9.17)	2.56 ± 0.122 (4.77)	1.30 ± 0.208 (15.91)
$\overline{\kappa_{EK}}$	11.93 ± 10.29 (8.63)	12.72 ± 24.16 (19.00)	13.29 ± 1.883 (14.16)	16.19 ± 1.921 (11.86)	13.92 ± 2.214 (15.90)	8.52 ± 1.719 (20.16)
κ_{lysis}^{He}	0.00 ± 0.00 (0.00)	0.00 ± 0.00 (0.00)	0.00 ± 0.000 (0.00)	0.00 ± 0.000 (0.00)	0.00 ± 0.000 (0.00)	0.00 ± 0.000 (0.00)
κ_{stres}^{He}	0.26 ± 0.00 (0.00)	0.26 ± 0.00 (0.00)	0.26 ± 0.000 (0.00)	0.00 ± 0.000 (0.00)	0.00 ± 0.000 (0.00)	0.00 ± 0.000 (0.00)
κ_{lysis}^{M}	0.00 ± 0.00 (0.00)	0.00 ± 0.00 (0.00)	0.00 ± 0.000 (0.00)	0.00 ± 0.000 (0.00)	0.00 ± 0.000 (0.00)	0.00 ± 0.000 (0.00)
κ_{stress}^{M}	0.00 ± 0.00 (0.00)	0.00 ± 0.00 (0.00)	0.00 ± 0.000 (0.00)	0.00 ± 0.000 (0.00)	0.00 ± 0.000 (0.00)	0.00 ± 0.000 (0.00)
Ψ	6.13 ± 4.02 (6.55)	0.00 ± 0.00 (0.00)	0.00 ± 0.00 (0.00)	0.00 ± 0.00 (0.00)	0.00 ± 0.00 (0.00)	0.00 ± 0.00 (0.00)
o	0.00 ± 0.00 (0.00)	0.13 ± 0.27 (20.95)	0.10 ± 0.013 (13.06)	8.86 ± 0.594 (6.71)	0.13 ± 0.014 (10.55)	0.04 ± 0.010 (23.34)
κ_{EK}^{P}	11.43 ± 9.59 (8.39)	1.26 ± 1.37 (10.89)	0.64 ± 0.083 (12.95)	8.10 ± 0.619 (7.64)	1.25 ± 0.064 (5.11)	0.32 ± 0.031 (9.79)
κ_{EM}^{He}	0.16 ± 0.09 (5.63)	0.12 ± 0.20 (16.84)	0.14 ± 0.021 (14.95)	0.18 ± 0.011 (6.35)	0.57 ± 0.011 (1.84)	0.08 ± 0.014 (18.03)
κ_{EM}^{M}	0.56 ± 0.09 (1.54)	0.84 ± 0.46 (5.44)	0.60 ± 0.029 (4.88)	0.20 ± 0.009 (4.49)	0.25 ± 0.016 (6.36)	0.05 ± 0.011 (23.30)

Supplementary Table 5: The estimated transition rate values of the MEK-SBM (multiple extracellular killing mechanisms of pathogens) with immune cell killing by pathogen-induced lysis for infection of WLA and R11 blood, respectively, with either *C. albicans*, *E. coli*, or *S. aureus*. The definition of each transition rate is given in Table 1 and in the caption of Table S5. The mean values and standard deviations (SD) of the transition rates were predicted by the global fitting procedure Metropolis Monte Carlo explained in the Methods section. The coefficient of variation (CV) refers to the ratio of the SD to the mean.

	mean × 10^{-2} $[min^{-1}]$ ± SD × 10^{-2} $[min^{-1}]$ (CV [%])					
**MEK-SBM + $\boldsymbol{\kappa_{lysis}^{IC}}$**	WLA chicken			R11 chicken		
Transition rate	*C. albicans*	*E. coli*	*S. aureus*	*C. albicans*	*E. coli*	*S. aureus*
Φ_{He}	1.74 ± 0.164 (9.44)	0.08 ± 0.011 (14.31)	0.22 ± 0.028 (12.73)	2.44 ± 0.313 (12.84)	0.62 ± 0.040 (6.45)	0.66 ± 0.048 (7.21)
Φ_{M}	10.84 ± 0.840 (7.76)	8.84 ± 0.860 (9.72)	6.08 ± 0.546 (8.98)	18.25 ± 2.706 (14.83)	2.94 ± 0.273 (9.30)	3.19 ± 0.157 (4.92)
κ_{M}	1.58 ± 0.240 (15.13)	4.20 ± 0.873 (20.79)	5.85 ± 0.747 (12.77)	3.03 ± 0.520 (17.15)	4.47 ± 0.564 (12.62)	6.12 ± 0.670 (10.94)
κ_{He}	11.89 ± 1.704 (14.34)	7.31 ± 1.689 (23.09)	6.44 ± 1.408 (21.87)	8.35 ± 0.607 (7.27)	5.34 ± 0.815 (15.27)	3.95 ± 0.623 (15.78)
ρ	0.67 ± 0.051 (7.59)	0.28 ± 0.037 (13.16)	0.07 ± 0.012 (17.03)	1.26 ± 0.241 (19.10)	0.48 ± 0.045 (9.51)	0.07 ± 0.004 (4.91)
γ	3.77 ± 0.332 (8.81)	3.95 ± 0.957 (24.25)	3.42 ± 0.523 (15.31)	2.38 ± 0.339 (14.27)	2.69 ± 0.621 (23.07)	2.41 ± 0.478 (19.85)
$\overline{\kappa_{EK}}$	10.51 ± 1.328 (12.63)	7.68 ± 1.654 (21.54)	21.58 ± 6.248 (28.96)	12.93 ± 1.073 (8.30)	8.29 ± 1.426 (17.21)	8.38 ± 1.442 (17.22)
κ_{lysis}^{He}	21.30 ± 2.098 (9.85)	17.70 ± 2.914 (16.46)	4.75 ± 0.949 (19.97)	27.61 ± 5.591 (20.25)	7.57 ± 0.711 (9.40)	2.76 ± 0.519 (18.83)
κ_{stres}^{He}	0.26 ± 0.000 (0.00)	0.26 ± 0.000 (0.00)	0.26 ± 0.000 (0.00)	0.00 ± 0.000 (0.00)	0.00 ± 0.000 (0.00)	0.00 ± 0.000 (0.00)
κ_{lysis}^{M}	33.65 ± 1.992 (5.92)	4.96 ± 0.713 (14.38)	4.33 ± 0.824 (19.01)	15.31 ± 1.923 (12.56)	5.12 ± 0.634 (12.39)	0.82 ± 0.149 (18.10)
κ_{stress}^{M}	0.00 ± 0.000 (0.00)	0.00 ± 0.000 (0.00)	0.00 ± 0.000 (0.00)	0.00 ± 0.000 (0.00)	0.00 ± 0.000 (0.00)	0.00 ± 0.000 (0.00)
Ψ	6.55 ± 0.665 (10.15)	0.00 ± 0.000 (0.00)	0.00 ± 0.000 (0.00)	0.00 ± 0.000 (0.00)	0.00 ± 0.000 (0.00)	0.00 ± 0.000 (0.00)
o	0.00 ± 0.000 (0.00)	0.09 ± 0.009 (10.67)	0.08 ± 0.011 (13.35)	3.48 ± 0.640 (18.39)	0.13 ± 0.018 (13.73)	0.05 ± 0.009 (17.88)
κ_{EK}^{P}	7.88 ± 0.619 (7.85)	1.50 ± 0.183 (12.19)	0.77 ± 0.088 (11.50)	7.52 ± 0.779 (10.37)	1.41 ± 0.066 (4.72)	0.36 ± 0.039 (11.04)
κ_{EM}^{He}	0.16 ± 0.013 (8.35)	0.10 ± 0.023 (23.09)	0.11 ± 0.018 (16.08)	0.18 ± 0.008 (4.61)	0.56 ± 0.011 (2.03)	0.06 ± 0.010 (16.01)
κ_{EM}^{M}	0.46 ± 0.025 (5.35)	0.77 ± 0.049 (6.44)	0.44 ± 0.032 (7.27)	0.11 ± 0.008 (7.05)	0.22 ± 0.029 (13.30)	0.04 ± 0.006 (15.48)

Supplementary Table 6: The estimated transition rate values of the MEK-SBM (multiple extracellular killing mechanisms of pathogens) without immune cell killing for infection of WLA and R11 blood, respectively, with either *C. albicans*, *E. coli*, or *S. aureus*. The definition of each transition rate is given in Table 1 and in the caption of Table S5. The mean values and standard deviations (SD) of the transition rates were predicted by the global fitting procedure Metropolis Monte Carlo explained in the Methods section. The coefficient of variation (CV) refers to the ratio of the SD to the mean.

	mean × 10^{-2} [min^{-1}] ± SD × 10^{-2} [min^{-1}] (CV [%])					
**MEK-SBM – κ_{EM}^{IC}**	WLA chicken			R11 chicken		
Transition rate	*C. albicans*	*E. coli*	*S. aureus*	*C. albicans*	*E. coli*	*S. aureus*
Φ_{He}	0.96 ± 0.061 (0.06)	0.09 ± 0.011 (11.93)	0.22 ± 0.023 (10.76)	1.58 ± 0.058 (3.67)	0.25 ± 0.018 (7.20)	0.48 ± 0.017 (3.54)
Φ_M	3.37 ± 0.167 (0.05)	3.48 ± 0.268 (7.69)	2.45 ± 0.064 (2.60)	12.30 ± 0.344 (2.80)	2.17 ± 0.101 (4.63)	2.93 ± 0.121 (4.11)
κ_M	3.41 ± 0.208 (0.06)	6.43 ± 0.585 (9.10)	7.31 ± 0.507 (6.93)	2.60 ± 0.100 (3.85)	4.77 ± 0.338 (7.10)	6.38 ± 0.600 (9.41)
κ_{He}	17.12 ± 2.444 (0.14)	13.24 ± 1.856 (14.02)	9.20 ± 2.082 (22.62)	11.64 ± 0.695 (5.97)	8.10 ± 1.124 (13.89)	6.01 ± 1.076 (17.90)
ρ	1.49 ± 0.066 (0.04)	0.92 ± 0.088 (9.49)	0.21 ± 0.017 (8.37)	0.73 ± 0.024 (3.32)	0.54 ± 0.044 (8.03)	0.12 ± 0.023 (18.77)
γ	5.38 ± 1.094 (0.20)	4.36 ± 0.942 (21.59)	2.11 ± 0.437 (20.71)	3.94 ± 0.216 (5.49)	2.44 ± 0.446 (18.29)	2.62 ± 0.580 (22.10)
$\overline{\kappa_{EK}}$	13.45 ± 1.417 (0.11)	8.93 ± 1.975 (22.13)	15.94 ± 2.999 (18.82)	13.96 ± 0.205 (1.47)	8.90 ± 1.201 (13.49)	9.29 ± 1.670 (17.97)
κ_{lysis}^{He}	0.00 ± 0.000 (0.00)	0.00 ± 0.000 (0.00)	0.00 ± 0.000 (0.00)	0.00 ± 0.000 (0.00)	0.00 ± 0.000 (0.00)	0.00 ± 0.000 (0.00)
κ_{stres}^{He}	0.26 ± 0.000 (0.00)	0.26 ± 0.000 (0.00)	0.26 ± 0.000 (0.00)	0.00 ± 0.000 (0.00)	0.00 ± 0.000 (0.00)	0.00 ± 0.000 (0.00)
κ_{lysis}^{M}	0.00 ± 0.000 (0.00)	0.00 ± 0.000 (0.00)	0.00 ± 0.000 (0.00)	0.00 ± 0.000 (0.00)	0.00 ± 0.000 (0.00)	0.00 ± 0.000 (0.00)
κ_{stress}^{M}	0.00 ± 0.000 (0.00)	0.00 ± 0.000 (0.00)	0.00 ± 0.000 (0.00)	0.00 ± 0.000 (0.00)	0.00 ± 0.000 (0.00)	0.00 ± 0.000 (0.00)
Ψ	9.49 ± 0.796 (0.08)	0.00 ± 0.000 (0.00)	0.00 ± 0.000 (0.00)	0.00 ± 0.000 (0.00)	0.00 ± 0.000 (0.00)	0.00 ± 0.000 (0.00)
o	0.00 ± 0.000 (0.00)	0.11 ± 0.026 (24.21)	0.08 ± 0.011 (14.34)	11.99 ± 0.364 (3.03)	0.14 ± 0.021 (15.05)	0.06 ± 0.007 (11.95)
κ_{EK}^{P}	19.91 ± 1.777 (0.09)	2.14 ± 0.136 (6.35)	0.82 ± 0.047 (5.68)	6.81 ± 0.132 (1.94)	1.48 ± 0.063 (4.25)	0.34 ± 0.046 (13.50)
κ_{EM}^{He}	0.00 ± 0.000 (0.00)	0.00 ± 0.000 (0.00)	0.00 ± 0.000 (0.00)	0.00 ± 0.000 (0.00)	0.00 ± 0.000 (0.00)	0.00 ± 0.000 (0.00)
κ_{EM}^{M}	0.00 ± 0.000 (0.00)	0.00 ± 0.000 (0.00)	0.00 ± 0.000 (0.00)	0.00 ± 0.000 (0.00)	0.00 ± 0.000 (0.00)	0.00 ± 0.000 (0.00)

Supplementary Table 7: The estimated transition rate values of the MEK-SBM (multiple extracellular killing mechanisms of pathogens) with immune cell killing by pathogen-induced lysis and without immune cell killing by extracellular mechanisms. The transition rate values were estimated for infection of samples from WLA and R11 chicken with *C. albicans* cells, *E. coli* cells or *S. aureus*. The definition of each transition rate is given in Table 1 and in the caption of Table S5. The mean values and standard deviations (SD) of the transition rates were predicted by the global fitting procedure Metropolis Monte Carlo explained in the Methods section. The coefficient of variation (CV) refers to the ratio of the SD to the mean.

	mean × 10^{-2} $[min^{-1}]$± SD × 10^{-2} $[min^{-1}]$ (CV [%])					
**MEK-SBM +$\boldsymbol{\kappa_{lysis}^{IC}} - \boldsymbol{\kappa_{EM}^{IC}}$**	WLA chicken			R11 chicken		
Transition rate	*C. albicans*	*E. coli*	*S. aureus*	*C. albicans*	*E. coli*	*S. aureus*
Φ_{He}	6.48 ± 0.434 (6.70)	0.22 ± 0.017 (7.57)	0.28 ± 0.037 (12.87)	3.98 ± 0.295 (7.40)	0.41 ± 0.047 (11.61)	0.66 ± 0.068 (10.39)
Φ_M	96.53 ± 2.127 (2.20)	49.55 ± 3.684 (7.43)	11.54 ± 0.758 (6.57)	36.95 ± 2.696 (7.30)	5.59 ± 0.288 (5.16)	3.08 ± 0.148 (4.81)
κ_M	1.19 ± 0.117 (9.77)	0.81 ± 0.088 (10.98)	1.42 ± 0.270 (19.08)	2.26 ± 0.308 (13.62)	1.07 ± 0.121 (11.32)	5.75 ± 0.678 (11.80)
κ_{He}	20.94 ± 1.886 (9.01)	7.47 ± 1.580 (21.15)	3.95 ± 0.726 (18.36)	5.78 ± 0.866 (14.98)	6.66 ± 1.417 (21.28)	3.82 ± 0.473 (12.40)
ρ	0.72 ± 0.044 (6.11)	0.27 ± 0.043 (16.01)	0.08 ± 0.016 (19.05)	0.75 ± 0.059 (7.87)	0.59 ± 0.047 (7.95)	0.08 ± 0.011 (14.44)
γ	3.14 ± 0.421 (13.41)	5.33 ± 0.678 (12.71)	1.59 ± 0.314 (19.79)	7.19 ± 1.113 (15.48)	3.71 ± 0.649 (17.47)	1.77 ± 0.306 (17.31)
$\overline{\kappa_{EK}}$	14.22 ± 1.090 (7.67)	6.43 ± 0.758 (11.79)	22.66 ± 2.079 (9.18)	16.48 ± 1.464 (8.88)	10.16 ± 0.985 (9.70)	12.55 ± 2.273 (18.11)
κ_{lysis}^{He}	12.13 ± 1.520 (12.53)	13.99 ± 2.780 (0.00)	15.13 ± 2.093 (0.00)	37.92 ± 3.575 (0.00)	15.24 ± 1.722 (0.00)	3.22 ± 0.428 (0.00)
κ_{stres}^{He}	0.26 ± 0.000 (0.00)	0.26 ± 0.000 (0.00)	0.26 ± 0.000 (0.00)	0.00 ± 0.000 (0.00)	0.00 ± 0.000 (0.00)	0.00 ± 0.000 (0.00)
κ_{lysis}^{M}	60.68 ± 5.122 (8.44)	28.08 ± 1.030 (0.00)	12.07 ± 0.659 (0.00)	34.52 ± 2.938 (0.00)	13.04 ± 1.054 (0.00)	0.85 ± 0.199 (0.00)
κ_{stress}^{M}	0.00 ± 0.000 (0.00)	0.00 ± 0.000 (0.00)	0.00 ± 0.000 (0.00)	0.00 ± 0.000 (0.00)	0.00 ± 0.000 (0.00)	0.00 ± 0.000 (0.00)
Ψ	0.96 ± 0.085 (8.79)	0.00 ± 0.000 (0.00)	0.00 ± 0.000 (0.00)	0.00 ± 0.000 (0.00)	0.00 ± 0.000 (0.00)	0.00 ± 0.000 (0.00)
o	0.00 ± 0.000 (0.00)	0.14 ± 0.018 (12.29)	0.08 ± 0.018 (24.03)	1.63 ± 0.097 (5.96)	0.13 ± 0.009 (6.62)	0.05 ± 0.010 (19.45)
κ_{EK}^{P}	7.67 ± 0.325 (4.24)	1.87 ± 0.107 (5.70)	1.11 ± 0.083 (7.44)	6.19 ± 0.245 (3.95)	1.67 ± 0.095 (5.66)	0.30 ± 0.039 (13.16)
κ_{EM}^{He}	0.00 ± 0.000 (0.00)	0.00 ± 0.000 (0.00)	0.00 ± 0.000 (0.00)	0.00 ± 0.000 (0.00)	0.00 ± 0.000 (0.00)	0.00 ± 0.000 (0.00)
κ_{EM}^{M}	0.00 ± 0.000 (0.00)	0.00 ± 0.000 (0.00)	0.00 ± 0.000 (0.00)	0.00 ± 0.000 (0.00)	0.00 ± 0.000 (0.00)	0.00 ± 0.000 (0.00)

Supplementary Table 8: Relation of phagocytosis rate by monocytes (Φ_M) and phagocytosis rate by heterophils (Φ_{He}) for all infection scenarios. The rates were predicted by calibrating the SEK- (single extracellular killing mechanism of pathogens) and the MEK-SBM (multiple extracellular killing mechanisms of pathogens) to experimental data of the corresponding infection scenario.

Φ_M / Φ_{He}		SEK-SBM	MEK-SBM
WLA chicken	*C. albicans*	2.6	5.8
	E. coli	89.8	101.7
	S. aureus	22.2	24.7
R11 chicken	*C. albicans*	4.9	7.8
	E. coli	3.8	5.4
	S. aureus	6.3	5.9

Supplementary Table 9: Relation of rates for phagocytosis by heterophils (Φ_{He}) between samples from R11 chicken and WLA chicken for bacterial infection with either *E. coli* cells or *S. aureus* cells. The rates were predicted by calibrating the SEK- (single extracellular killing mechanism of pathogens) and the MEK-SBM (multiple extracellular killing mechanisms of pathogens) to experimental data of the corresponding infection scenario.

$\Phi_{He}^{R11} / \Phi_{He}^{WLA}$	SEK-SBM	MEK-SBM
E. coli	5.4	6.9
S. aureus	2.3	2.7

Supplementary Table 10: Relation of rates for monocyte killing (κ_{EM}^{M}) between samples from WLA chicken and R11 chicken for infection with either *C. albicans* cells, *E. coli* cells or *S. aureus* cells. The rates were predicted by calibrating the SEK- (single extracellular killing mechanism of pathogens) and the MEK-SBM (multiple extracellular killing mechanisms of pathogens) to experimental data of the corresponding infection scenario.

$\kappa_{EM}^{M}(WLA) / \kappa_{EM}^{M}(R11)$	SEK-SBM	MEK-SBM
C. albicans	2.5	2.8
E. coli	3.9	3.4
S. aureus	11.3	12.4

Supplementary Table 11: Relation of rates for monocyte killing ($\boldsymbol{\kappa_{EM}^{M}}$) and heterophil killing ($\boldsymbol{\kappa_{EM}^{He}}$) between *E. coli* injection and *C. albicans* or *S. aureus* injection into samples from R11 chickens. The rates were predicted by calibrating the SEK- (single extracellular killing mechanism of pathogens) and the MEK-SBM (multiple extracellular killing mechanisms of pathogens) to experimental data of the corresponding infection scenario.

		SEK-SBM	MEK-SBM
κ_{EM}^{M}	$\kappa_{EM}^{M}(E.coli)$ / $\kappa_{EM}^{M}(C.albicans)$	1.4	1.2
	$\kappa_{EM}^{M}(E.coli)$ / $\kappa_{EM}^{M}(S.aureus)$	5.3	5.1
κ_{EM}^{He}	$\kappa_{EM}^{He}(E.coli)$ / $\kappa_{EM}^{He}(C.albicans)$	2.7	3.2
	$\kappa_{EM}^{He}(E.coli)$ / $\kappa_{EM}^{He}(S.aureus)$	9.6	7.3
