# Supplementary material for: Dynamic Interplay of Host and Pathogens in an Avian Whole-Blood Model
Source: Front Immunol. 2020 Mar 31;11:500. doi: 10.3389/fimmu.2020.00500 (PMC7136455; doi:10.3389/fimmu.2020.00500)

Supplementary Material

1 Supplementary Methods

1.1 Real-time reverse transcription (RT)-PCR

The following primers were used for RT-PCR in this study:

Avian RNA target and primer description		Sequence (5′-3′)	NCBI accession number
GAPDH	Forward	GTCAGCAATGCATCGTGCA	K01458
	Reverse	GGCATGGACAGTGGTCATAAGA	
IFN γ	Forward	CCCGATGAACGACTTGAGAAT	NM_205149
	Reverse	AGACTGGCTCCTTTTCCTTTTG	
IL-1β	Forward	CAGCCAGAAAGTGAGGCTCAA	NM_204524
	Reverse	CGCTCATCACACACGACATGTA	
IL-6	Forward	TTCGACGAGGAGAAATGCCT	NM_204628
	Reverse	CGACGTTCTGCTTTTCGCTAT	
IL-8	Forward	AAGGCACTTATGGCCAAGGCT	NM_205498
	Reverse	ACCGATGTGGAAGGTGGAAGA	
iNOS	Forward	GAACAGCCAGCTCATCCGATA	AY648162
	Reverse	CCCAAGCTCAATGCACAACTT	
K60	Forward	CACAGAACCAAACCCAGGTGA	NM_205018
	Reverse	AGCCATACCTTTTGCTCCAGC	
LITAF	Forward	GCTGTTCTATGACCGCCCAGTT	NM_204267
	Reverse	AACAACCAGCTATGCACCCCA	
MIP1β	Forward	TCCTGCTGCTTCACCTACATCT	AJ243034
	Reverse	ATGAACACAACACCAGCATGAG	

GAPDH was used as housekeeping gene for normalization of gene expression. Expression of GAPDH has been shown previously to be unaltered in immune cells during infection with *Salmonella* and in intestinal tissue ((1) and Jana Pieper, Dissertation; https://www.db-thueringen.de/receive/dbt_mods_00012048). The primer efficacy was tested in previous studies using

various sample types, such as chicken caecum (2), isolated immune cells (Jana Pieper, Dissertation; https://www.db-thueringen.de/receive/dbt_mods_00012048), and various embryonic tissues (3).

RNA was isolated from 100 µl whole blood of each animal, infection, and time point, and aliquots of the eluted RNA were used for subsequent RT-PCR. Due to variations in leucocyte numbers (see Supplementary Table 1 and Figure 2) resulting in variations in RNA content, some variation was expected and observed for GAPDH Ct values (mean ± SD):

min after infection	non-infected	*C. albicans*	*S. aureus*	*E. coli*
30	31.31 ± 0.99	33.63 ± 0.92	30.96 ± 0.98	33.74 ± 1.63
90	32.55 ± 1.66	34.42 ± 0.96	29.78 ± 1.11	33.16 ± 1.35
150	32.85 ± 1.66	31.99 ± 0.78	30.82 ± 1.14	34.22 ± 1.51
240	31.22 ± 1.29	35.23 ± 1.79	32.11 ± 1.43	34.61 ± 1.44

1.2 Model Parameter Estimation

In order to estimate the values of the *a priori* unknown transition rate, we applied the method *Simulated Annealing* based on *Metropolis Monte Carlo* [REF: Kirkpatrick 1983]. This global parameter estimation algorithm is able to find optimal model parameter values that yield the best fit of the simulated model kinetics to experimental measurements. The algorithm performs a random walk through the multi-dimensional parameter space. At each position on this walk, the set of model parameters $\vec{p}$ is scored by calculating the distance of the model simulation with $\vec{p}$ to the experimental data. This is performed by calculating the weighted sum of the least squares errors (LSE)

$$\mathrm{E}(\vec{p}) = \sum_c \omega_c \varepsilon_c[\vec{p}]\,, \tag{1}$$

where the weight $\omega_c$is specific for each combined unit c. The least squares error (LSE) ε_c is given by

$$\varepsilon[\vec{p}] = \frac{1}{2}\sum_i \left(x_{i,c}^{exp} - x_{i,c}^{sim}(\vec{p})\right)^2 \tag{2}$$

and is defined as the sum of the squared difference between the experimental data ($x_{i,c}^{exp}$) and the comparable simulated data ($x_{i,c}^{sim}$), which forms the combined unit c, for each time point i. The definition of each combined unit is provided in the main manuscript in section 2.8. Mathematical Modeling.

Based on the current position $\vec{p}$ in the parameter space, the next potential position $\overrightarrow{p'}$ is calculated by varying $\vec{p}$ in the range of 0 to 10%. Whether this parameter set is accepted and thereby, this step will

be taken is dependent on the corresponding score of $\overrightarrow{p'}$. If this score $E'[\vec{p}]$ is smaller than the score of the current parameter set $\vec{p}$, $E[\vec{p}]$, *i.e.* $\Delta E = E'[\vec{p}] - E[\vec{p}] < 0$, then the new parameter set $\overrightarrow{p'}$ is accepted and the next step to the new position will be performed by $\vec{p} \leftarrow \overrightarrow{p'}$. This initiates a new fitting step. However, if $\Delta E > 0$, *i.e.* $E'[\overrightarrow{p'}]$ the score of the potential parameter set $\overrightarrow{p'}$ is larger than the score current parameter set $E[\vec{p}]$, than the algorithm continues with the Metropolis step, where the potential parameter set $\overrightarrow{p'}$ will be accepted if

$$e^{-\tau(f)\Delta E} > r. \quad (3)$$

Here, $r \in [0,1]$ is a uniformly distributed random number that and $\tau(f)$ plays the role of the inverse 'system temperature'. If the annealing process starts and the temperature decreases with continuing fitting steps f, the inverse temperature $\tau(f)$ increases according the Hill function

$$\tau(f) = \tau_0 + (\tau_\infty - \tau_0)\, \frac{f^n}{K^n + f^n}\ , \quad (4)$$

where the Hill exponent n and the parameter K denote the smooth increase of $\tau(f)$ from τ_0 to τ_∞ with increasing f. Thereby, the acceptance probability of worse parameter sets, *i.e.* those with a larger score, decrease with continuing fitting steps. By accepting worse parameters by means of the Metropolis criterion, local minima can be passed and the global minimum can be found.

In order to increase the statistical robustness of this procedure, we repeated the whole procedure starting at randomly chosen positions in the parameter space and calculated the mean and the standard deviations. Furthermore, we performed the fitting procedure for different system sizes. We stepwise increased the number of cells in the system by factor 10 until the real system size is reached. Since the more realistic system sizes are associated with increasing computational effort, we adapted the number of fitting steps to avoid computational overload. The settings of the estimation algorithm for the different system sizes are provided in Supplementary Material.

1.3 Model comparison based on Akaike Information Criterion

The different mathematical models of avian whole-blood infection were compared using the Akaike information criterion (4). This score aims at ranking the different models by not only including the agreement of the models with experimental data, like the LSE (see Eq 8), but also by incorporating the complexity of the models regarding the number of parameters. As described in Burnham *et al.* 2014 (5), in the special case of least squares estimation, the AIC is defined by

$$AIC = n \ln\left(\frac{\hat{\sigma}^2}{n}\right) + 2K. \quad (5)$$

Here, the second term represents the penalty by the number of model parameters K, which increases proportionally with K. The first term of Eq. 5 determines the influence by the number of independent experimental data points n and the agreement of the model with the experimental data by the sum of squared residuals

$$\hat{\sigma} = \sum_{i=1}^{i=n}\left(x_i^{dat} - x_i^{sim}\left[\vec{p}_{opt}\right]\right)^2. \qquad (6)$$

This variable is calculated by sum of the squared differences between experimental data (x^{dat}) and simulated data ($x^{dat}\left[\vec{p}_{opt}\right]$) resulting from simulating the model with the optimal parameter set $\vec{p}_{opt}$ at each of the ith experimentally measured data points ($i = 1, 2, 3, \dots, 48$). If the number of experimental data points n is small relative to the number of model parameters K, *i.e.* when $\frac{n}{K} \leq 40$, then the corrected version of the AIC, the

$$AIC_c = AIC + \frac{2K(K+1)}{n-K-1} \qquad (7)$$

must be applied (5). After calculating the AIC_c value for each of the m models, the models were ranked according a relative measure the

$$\Delta_i = AIC_c^i - AIC_c^{min}. \qquad (8)$$

Here, the relative AIC_c distance of the ith model with AIC_c^i to the best model, *i.e.* the model with the smallest AIC_c (AIC_c^{min}), is calculated.

This distance is used to categorize the models by means of the following assessment guidelines (6)

$$C(\Delta_i) = \begin{cases} \Delta_i \leq 2, & C = model\ i\ has\ substantial\ support \\ 4 \leq \Delta_i \leq 7, & C = model\ i\ has\ considerably\ less\ support \\ \Delta_i > 10, & C = model\ i\ has\ essentially\ no\ support \end{cases}$$

References

1. Jawale CV, Lee JH. Characterization of a *Salmonella* Typhimurium ghost carrying an adjuvant protein as a vaccine candidate for the protection of chickens against virulent challenge. Avian Pathol. 2014;43(6):506-13.

2. Berndt A, Wilhelm A, Jugert C, Pieper J, Sachse K, Methner U. Chicken cecum immune response to *Salmonella enterica* serovars of different levels of invasiveness. Infect Immun. 2007;75(12):5993-6007.

3. Braukmann M, Sachse K, Jacobsen ID, Westermann M, Menge C, Saluz HP, et al. Distinct intensity of host-pathogen interactions in Chlamydia psittaci- and Chlamydia abortus-infected chicken embryos. Infect Immun. 2012;80(9):2976-88.

4. Akaike H. New Look at Statistical-Model Identification. IEEE Transactions on Automatic Control. 1974;AC19:716-23.

5. Burnham K, Anderson D. Multimodel Inference: understanding AIC and BIC in Model Selection. Sociological Methods Research. 2004;33:261-304.

6. Burnham K, Anderson D. Model Selection and Multimodel Inference: A Practical Information-Theoretic Approach2002.

2 Supplementary Figures and Tables

2.1 Supplementary Figures

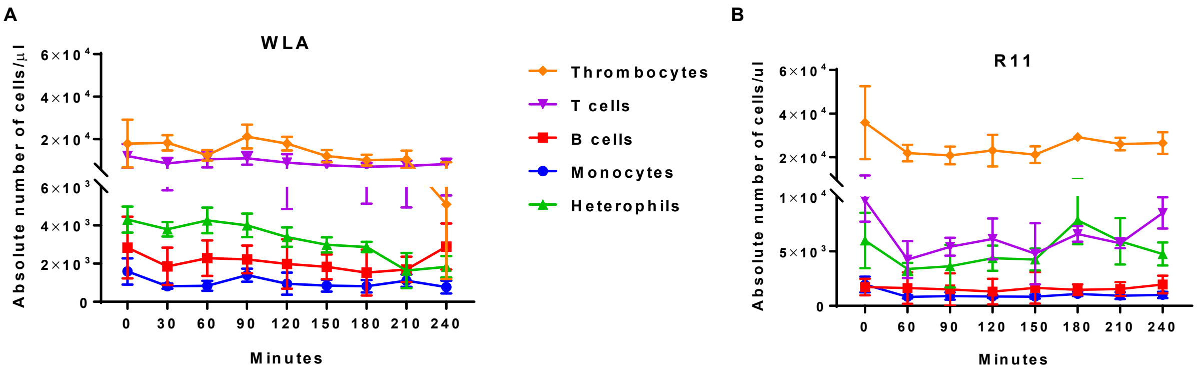

Supplementary Figure 1. Absolute numbers of immune cells in non-infected blood samples. Absolute numbers of viable monocytes, heterophils, thrombocytes, T and B cells were determined every 30 to 60 min in whole blood collected from WLA chickens (A) and R11 chickens (B) using flow cytometry. Data of six independent experiments using blood from different donors is presented as mean and SD.

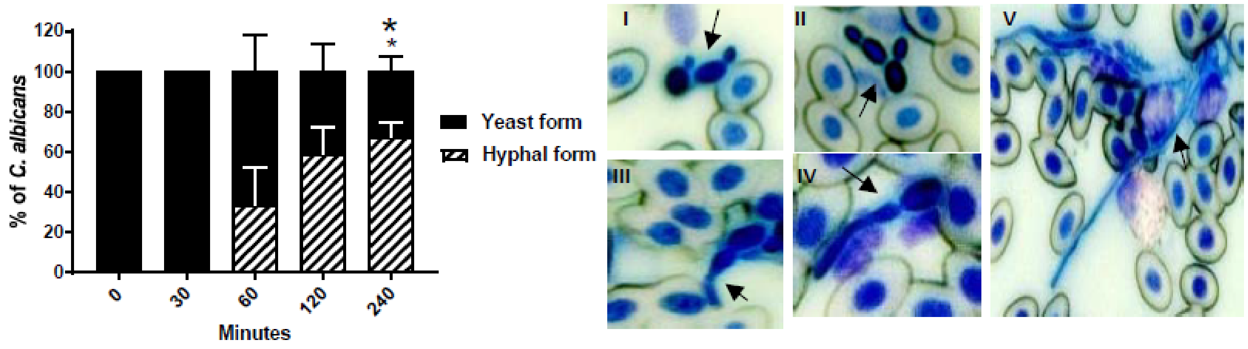

Supplementary Figure 2. Hyphae development of *C. albicans* in chicken blood. Fungal morphology was assessed in Giemsa stained blood smears. (A) Quantification of morphology, data of six independent experiments using blood from different donors presented as mean and SD. (B) Representative micrographs showing yeast cells after 0 and 30 min (I, II), germ tube formation after 60 min (III) and hyphae after 120 and 240 min (IV, V).

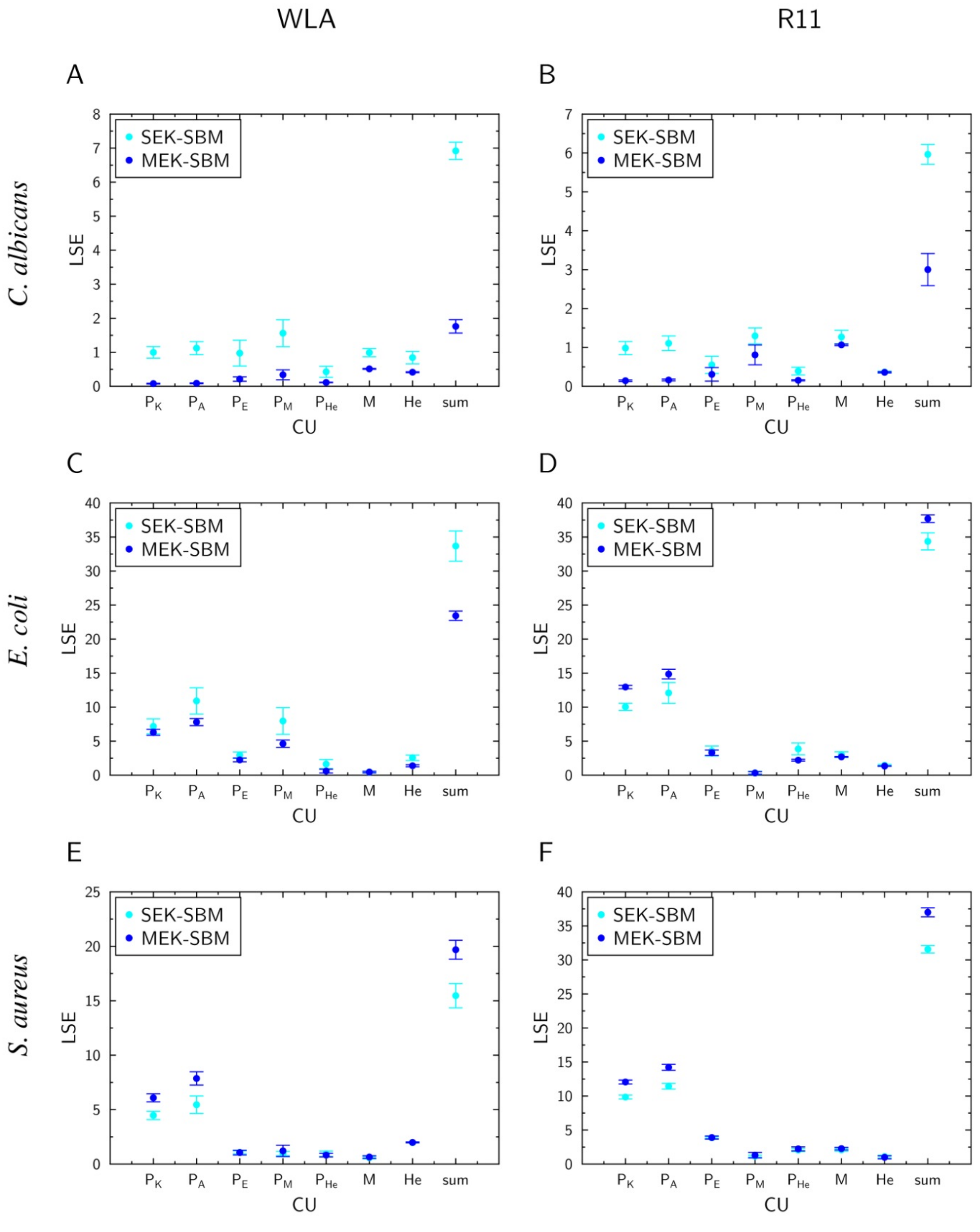

Supplementary Figure 3: Least squares error (LSE) for model simulations by the SEK-SBM and the MEK-SBM. The LSE between experimental data and simulated data of the SEK-SBM and the MEK-SBM simulation for the combined units (CUs) of killed pathogens (P_K), alive pathogens (P_A), extracellular pathogens (P_E), pathogens in monocytes (P_M), pathogens in heterophils (P_{He}), the number of monocytes (M) and heterophils (He). The models were calibrated to experimental data of whole-blood samples from WLA chicken (left column) and R11 chicken (right column) that were infected with *C. albicans* (A, B), *E. coli* (C, D), and *S. aureus* (E, F).

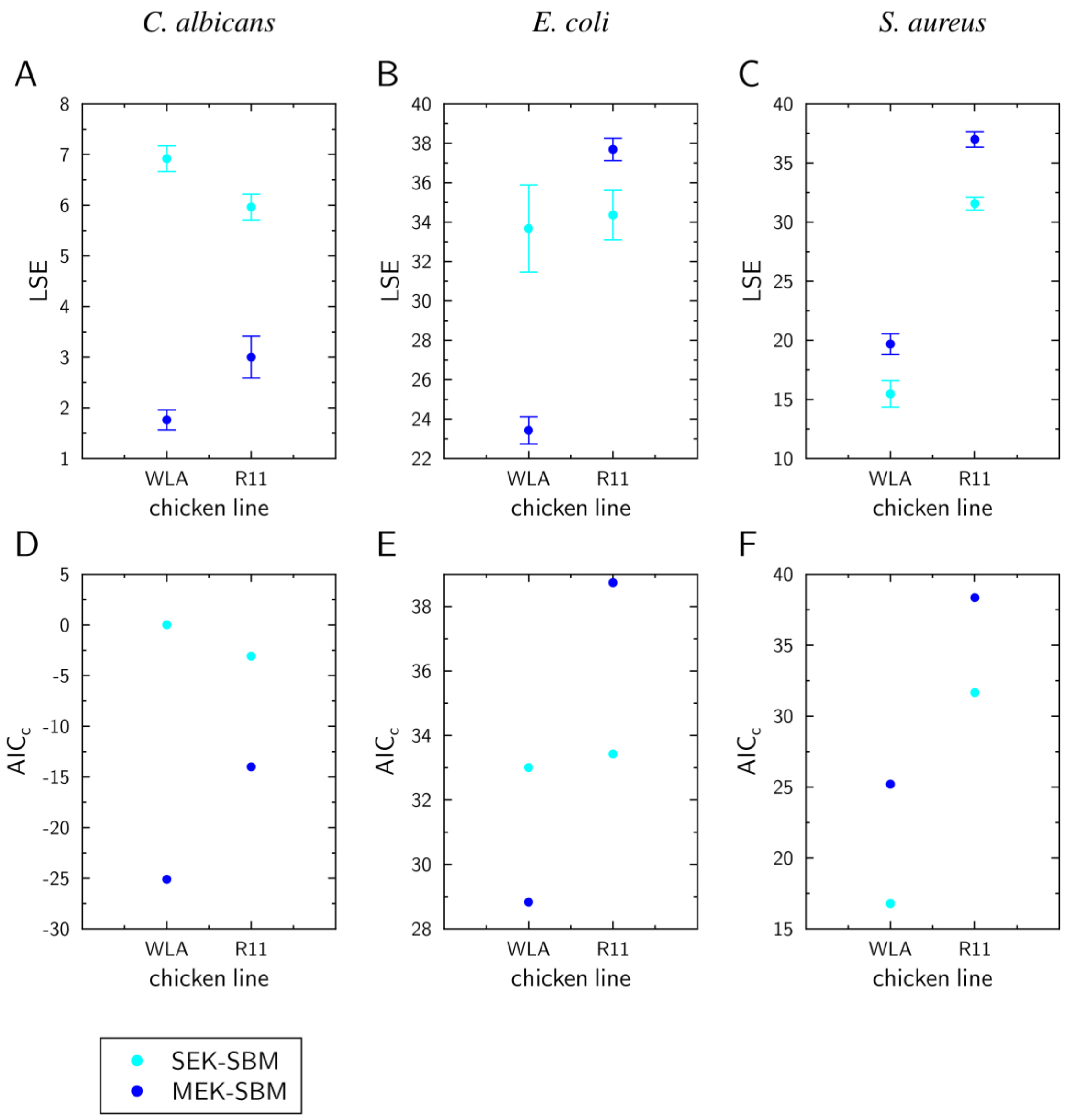

Supplementary Figure 4: Total least squares error (LSE) and corrected Akaike information criterion (AIC_C) of the SBMs for different infection scenarios. The LSE scores the agreement between experimental data and simulated data by the SEK-SBM (single extracellular killing mechanism of pathogens) and the MEK-SBM (multiple extracellular killing mechanisms of pathogens). The data points and error bars depict the mean $\pm$ standard deviation of simulation results observed from 50 simulations for normally distributed transition rates. The model simulations were calibrated to experimental data of whole-blood infection of samples from WLA and R11 chicken with *C. albicans* (A), *E. coli* (B) and *S. aureus* (C). The corrected AIC (AIC_C) additionally involves the model complexity of the SBMs and is calculated for simulations of whole-blood infection with *C. albicans* (D), *E. coli* (E) and *S. aureus* (F).

Supplementary Figure 5: Comparison of kinetics of alive pathogens simulated by the SEK-SBM (single extracellular killing mechanism of pathogens) and the MEK-SBM (multiple extracellular killing mechanisms of pathogens). Model simulations (solid lines) of alive *C. albicans* (A, B), *E. coli* (C, D) and *S. aureus* (E, F) that were injected into whole-blood samples from WLA chickens (left column) and R11 chickens (right column). The thickness of the solid lines represents the mean ± standard deviation of simulation results observed from 50 simulations for normally distributed transition rates. Experimental data are represented by black data points that are connected by dashed lines as guide for the eye.

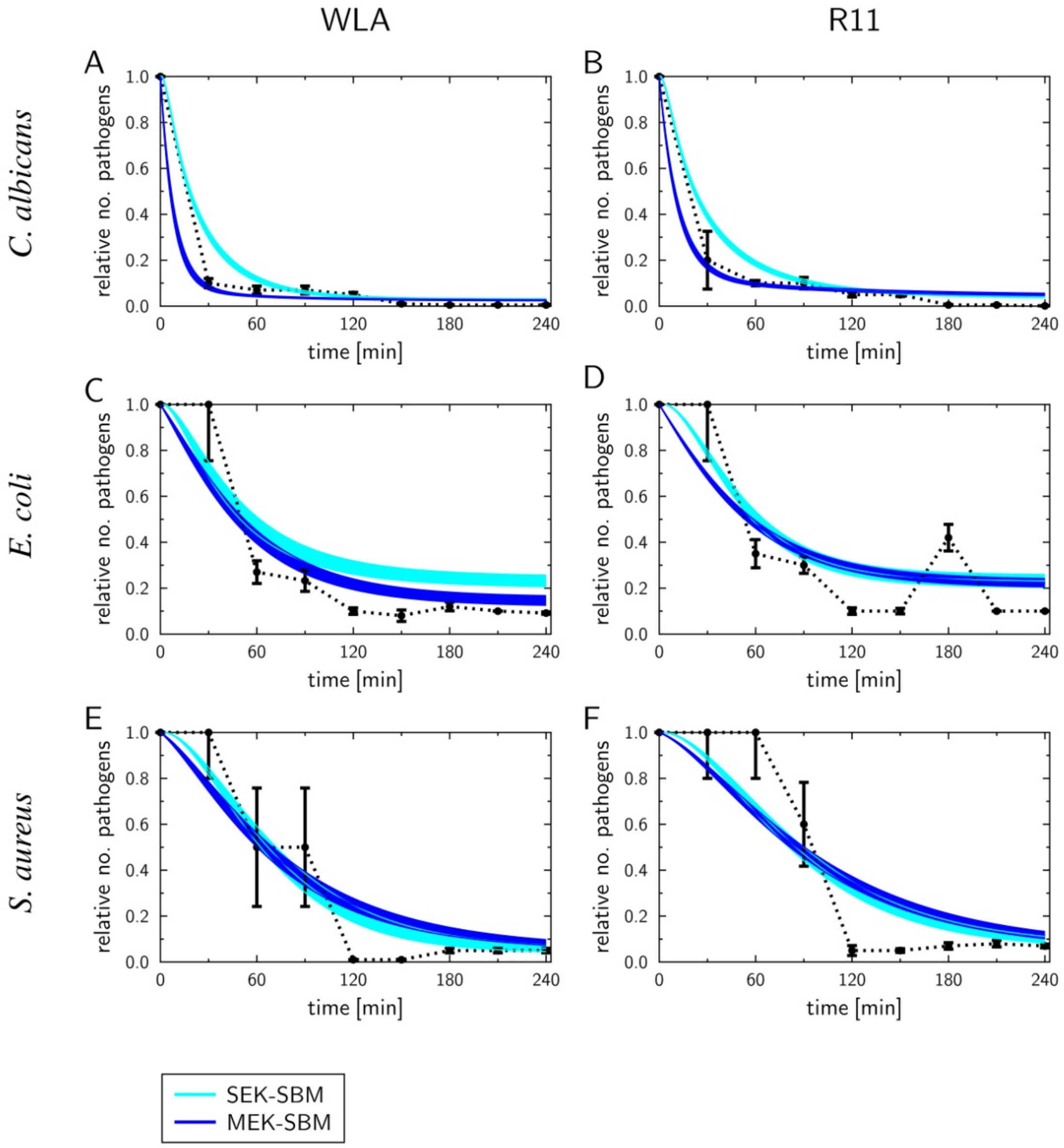

Supplementary Figure 6: Time course of the rate for extracellular killing by antimicrobial peptides predicted by the SEK-SBM (single extracellular killing mechanism of pathogens) and the MEK-SBM (multiple extracellular killing mechanisms of pathogens). The thickness of the solid lines represents the mean ± standard deviation of simulation results observed from 50 simulations for normally distributed transition rates. These rates were predicted by calibrating the models to experimental data of whole-blood samples from WLA chickens (left column) and R11 chickens (right column) that were infected with *C. albicans* (A, B), *E. coli* (C, D) and *S. aureus* (E, F).

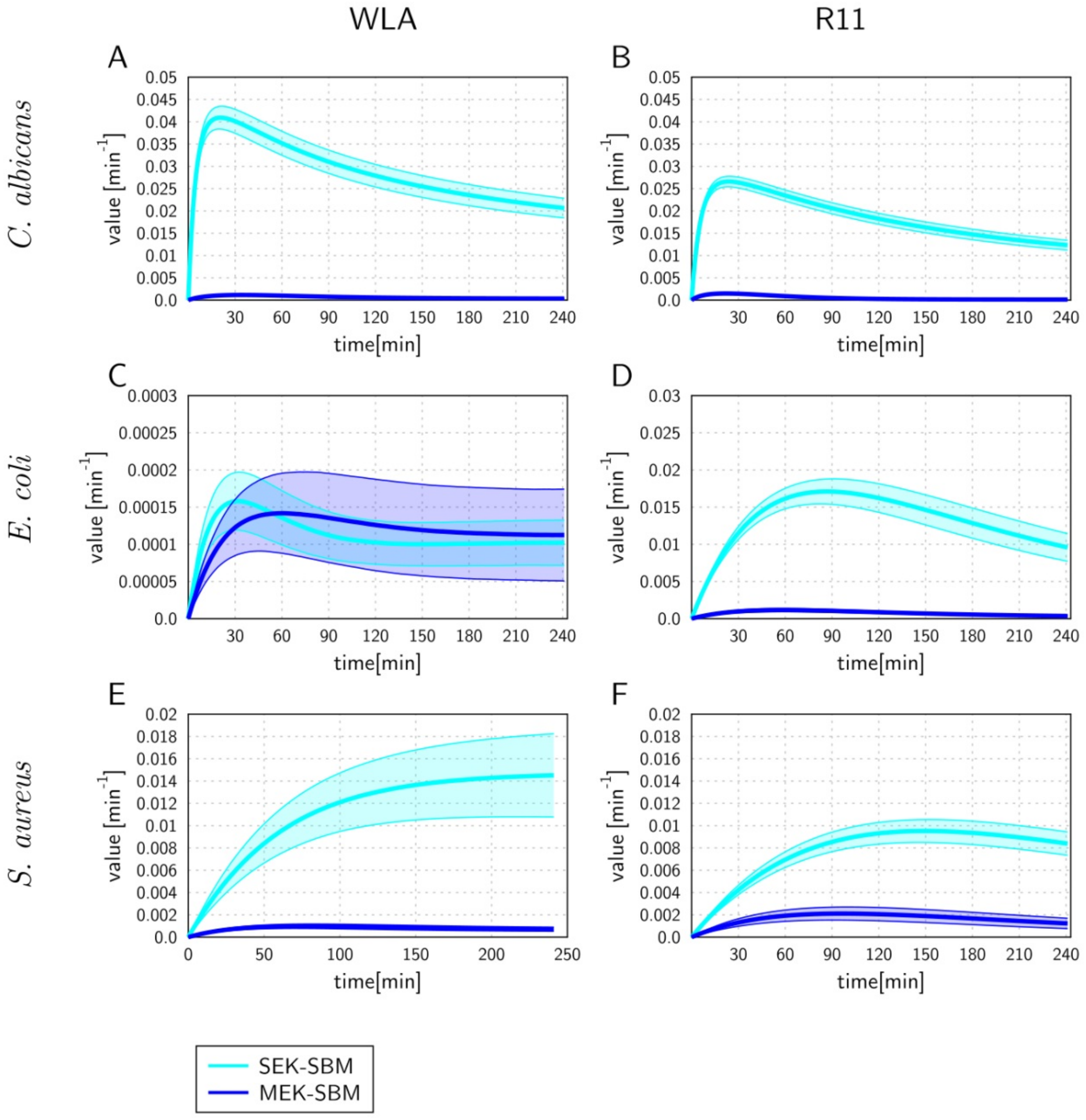

Supplementary Figure 7: Comparison of kinetics of pathogens in heterophils simulated by the SEK-SBM (single extracellular killing mechanism of pathogens) and the MEK-SBM (multiple extracellular killing mechanisms of pathogens). Model simulations (solid lines) of infection scenarios with *C. albicans* cells (A, B), *E. coli* cells (C, D) and *S. aureus* cells (E, F) that were injected into whole-blood samples from WLA chickens (left column) and R11 chickens (right column). The thickness of the solid lines represents the mean ± standard deviation of simulation results observed from 50 simulations for normally distributed transition rates. Experimental data are represented by black data points that are connected by dashed lines as guide for the eye.

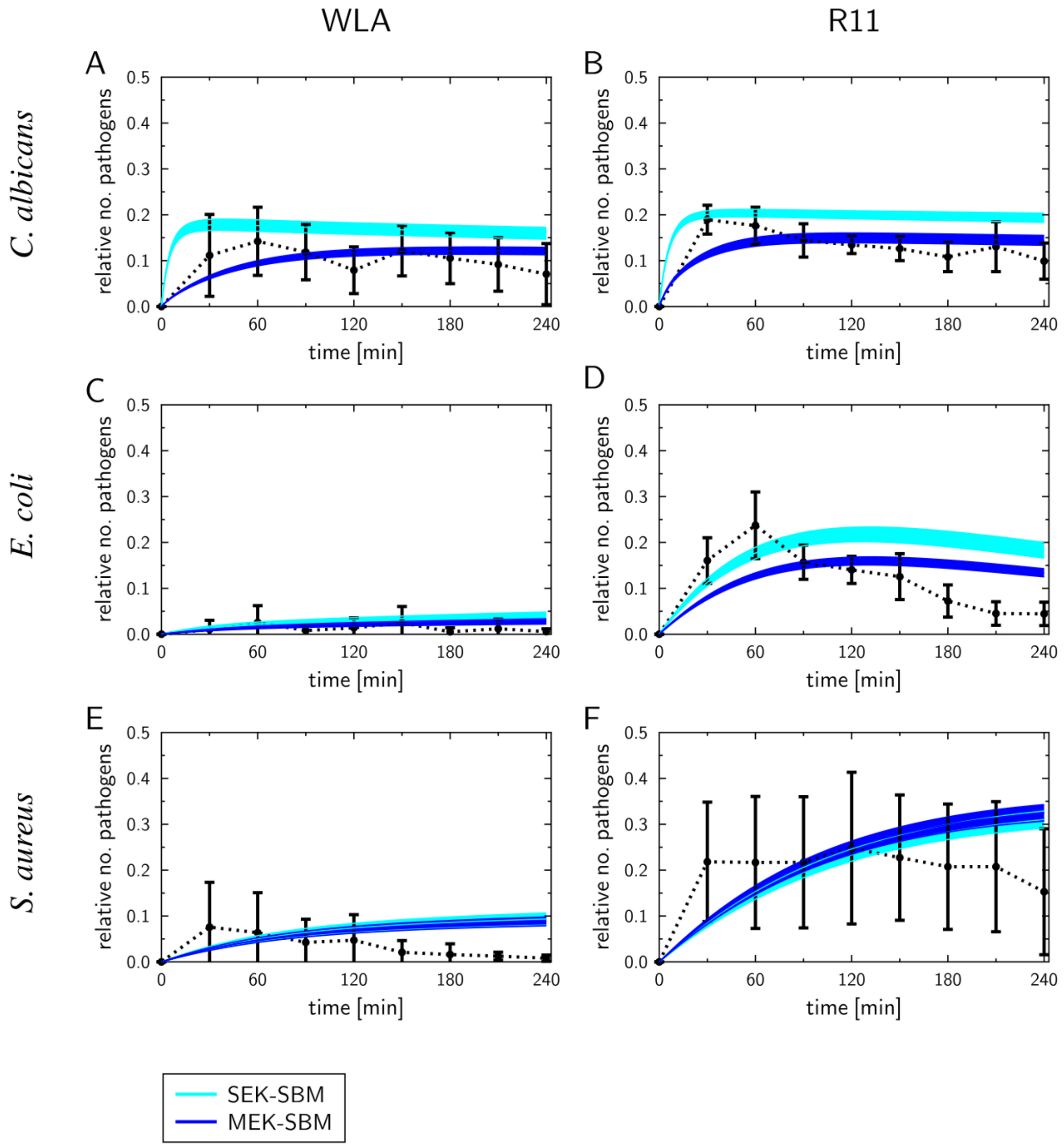

Supplementary Figure 8: Rates for phagocytosis of pathogens by heterophils (Φ_{He}) and monocytes (Φ_M) predicted by the SEK-SBM (single extracellular killing mechanism of pathogens) and the MEK-SBM (multiple extracellular killing mechanisms of pathogens). The models were calibrated to experimental data of infection scenarios with *C. albicans* cells (left column), *E. coli* cells (middle column) and *S. aureus* cells (right column) that were injected into whole-blood samples from WLA chickens (A-C) and R11 chickens (D-E).

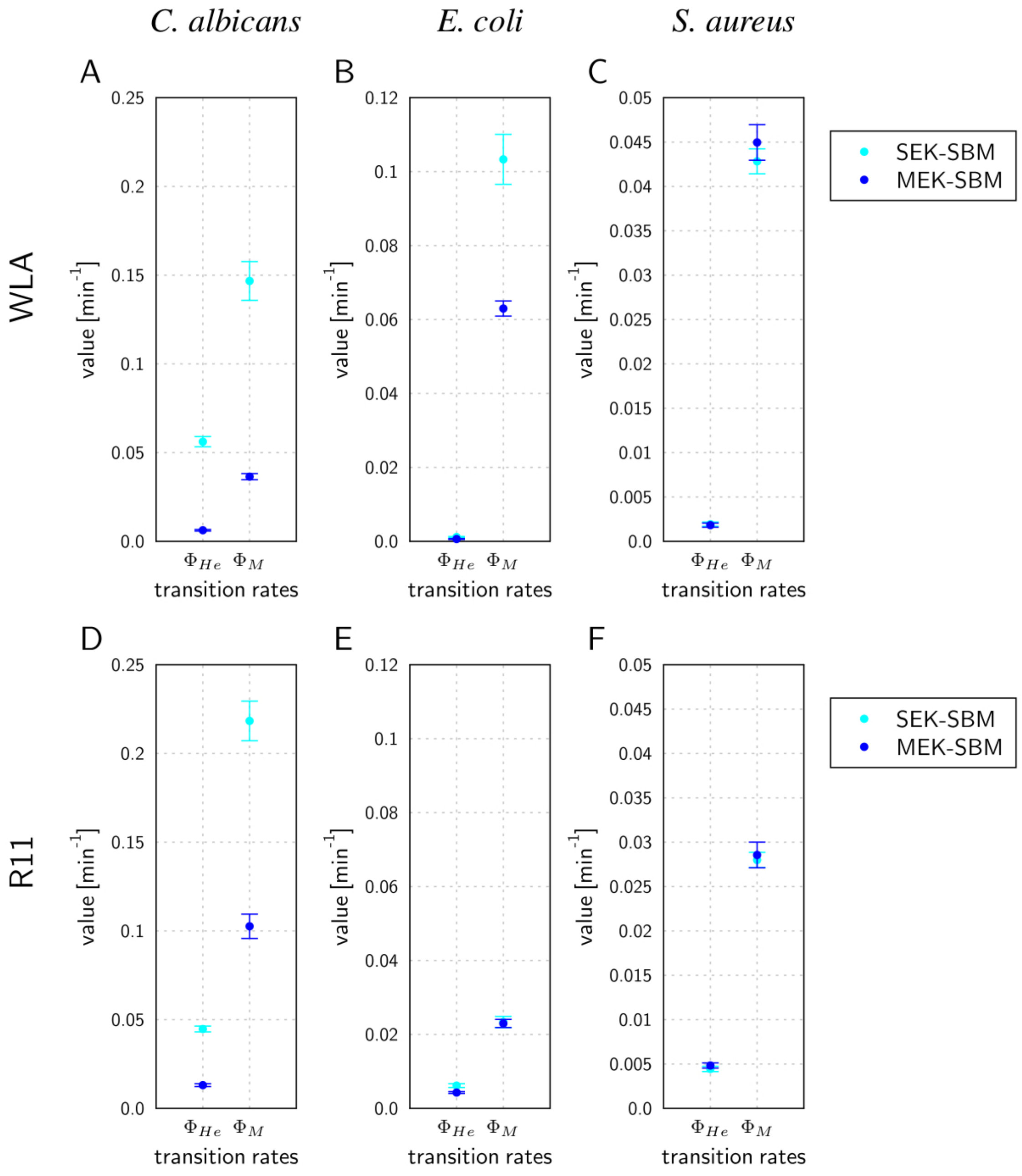

Supplementary Figure 9: Comparisons of kinetics of pathogens in monocytes simulated by the SEK-SBM (single extracellular killing mechanism of pathogens) and the MEK-SBM (multiple extracellular killing mechanisms of pathogens). Model simulations (solid lines) of infection scenarios with *C. albicans* cells (A, B), *E. coli* cells (C, D) and *S. aureus* cells (E, F) that were injected into whole-blood samples from WLA chickens (left column) and R11 chickens (right column). The thickness of the solid lines represents the mean ± standard deviation of simulation results observed from 50 simulations for normally distributed transition rates. Experimental data are represented by black data points that are connected by dashed lines as guide for the eye.

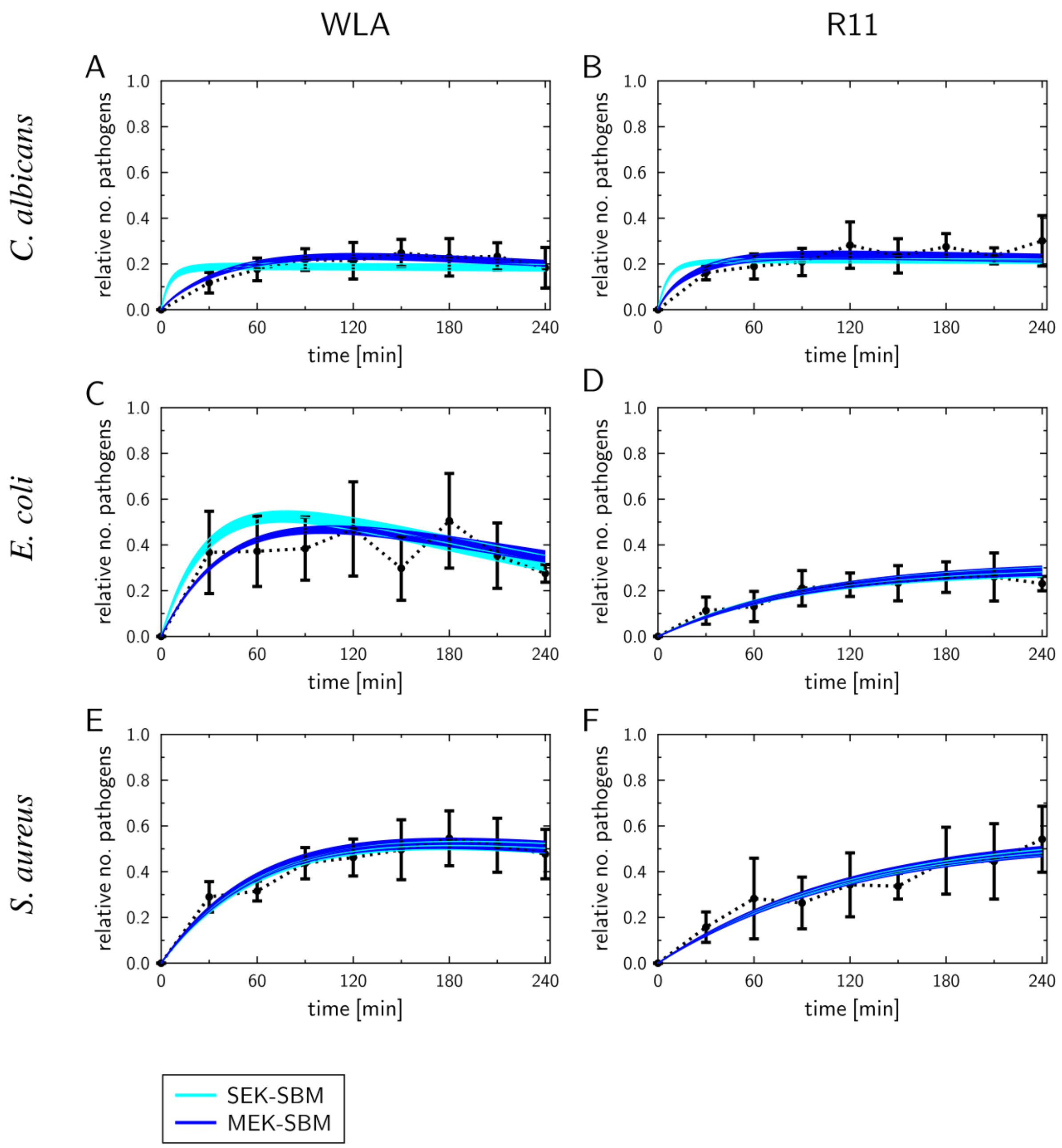

Supplementary Figure 10: Comparisons of kinetics of monocytes simulated by the SEK-SBM (single extracellular killing mechanism of pathogens) and the MEK-SBM (multiple extracellular killing mechanisms of pathogens). Model simulations (solid lines) of infection scenarios with *C. albicans* cells (A, B), *E. coli* cells (C, D) and *S. aureus* cells (E, F) that were injected into whole-blood samples from WLA chickens (left column) and R11 chickens (right column). The thickness of the solid lines represents the mean ± standard deviation of simulation results observed from 50 simulations for normally distributed transition rates. Experimental data are represented by black data points that are connected by dashed lines as guide for the eye.

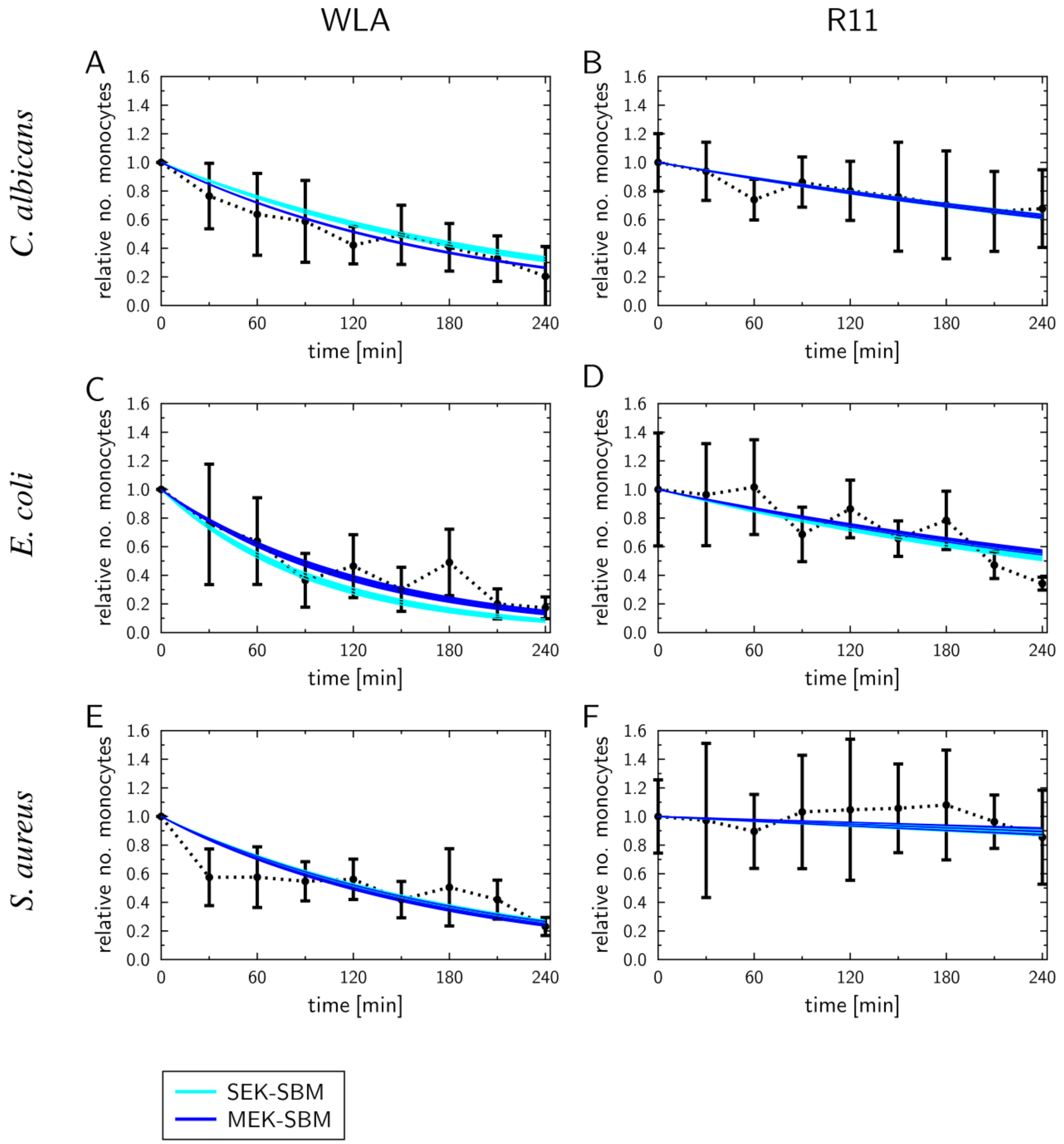

Supplementary Figure 11: Comparisons of kinetics of heterophils simulated by the SEK-SBM (single extracellular killing mechanism of pathogens) and the MEK-SBM (multiple extracellular killing mechanisms of pathogens). Model simulations (solid lines) of infection scenarios with *C. albicans* cells (A, B), *E. coli* cells (C, D) and *S. aureus* cells (E, F) that were injected into whole-blood samples from WLA chickens (left column) and R11 chickens (right column). The thickness of the solid lines represents the mean ± standard deviation of simulation results observed from 50 simulations for normally distributed transition rates. Experimental data are represented by black data points that are connected by dashed lines as guide for the eye.

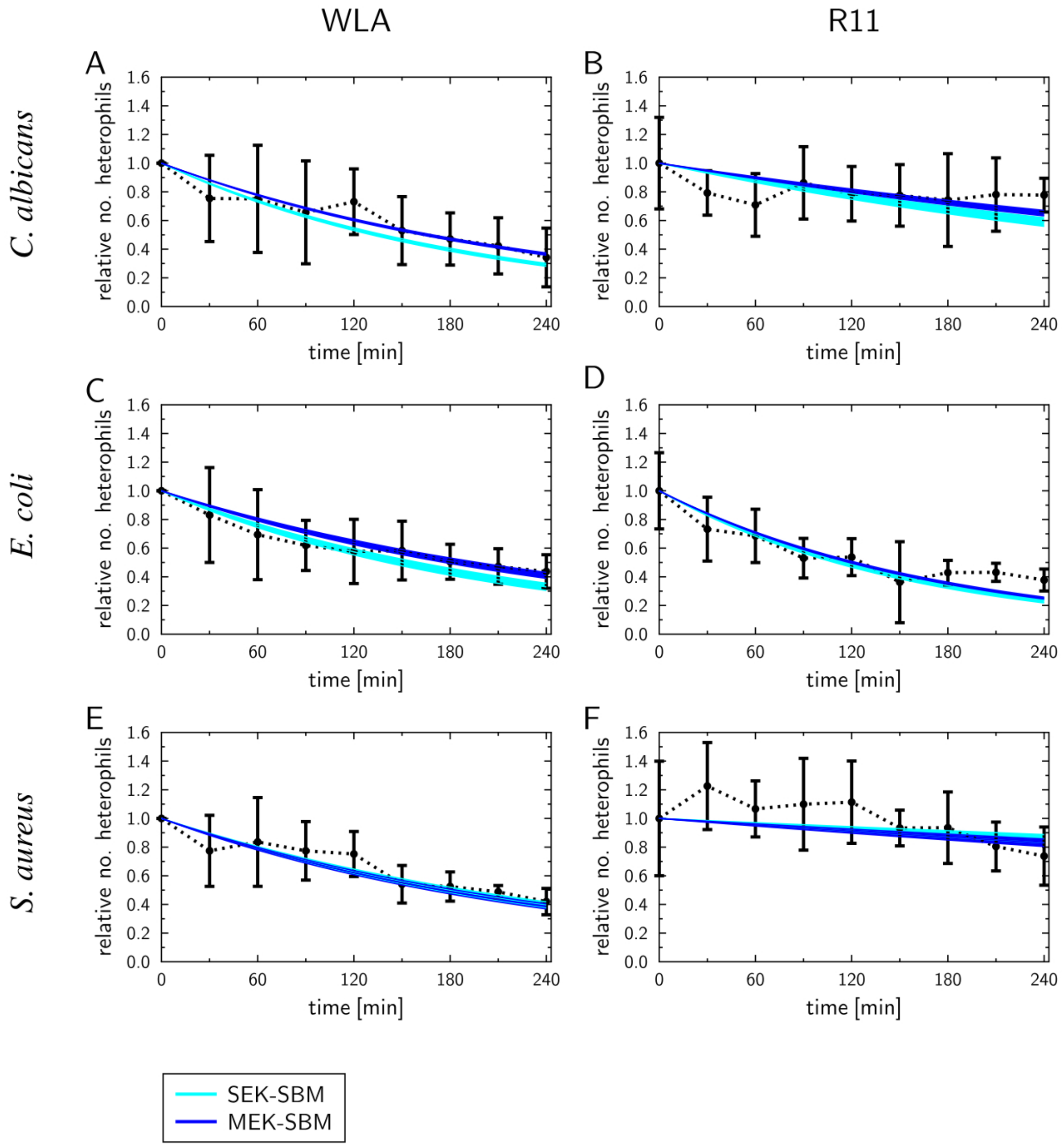

Supplementary Figure 12: Time course of the heterophil number during infection simulated by the MEK-SBM without immune cell killing by extracellular mechanisms induced by pathogens with rate κ_{EM}^{He} for heterophils and rate κ_{EM}^{M} for monocytes. The model was calibrated to experimental data on whole-blood infection of samples from WLA chickens (A) and R11 chickens (B). Experimental data are represented by data points that were connected by dotted lines as guide for the eye. The resulting decrease of heterophils in WLA chicken is caused by killing due to the experimental setup with rate κ_{stress}^{He}.

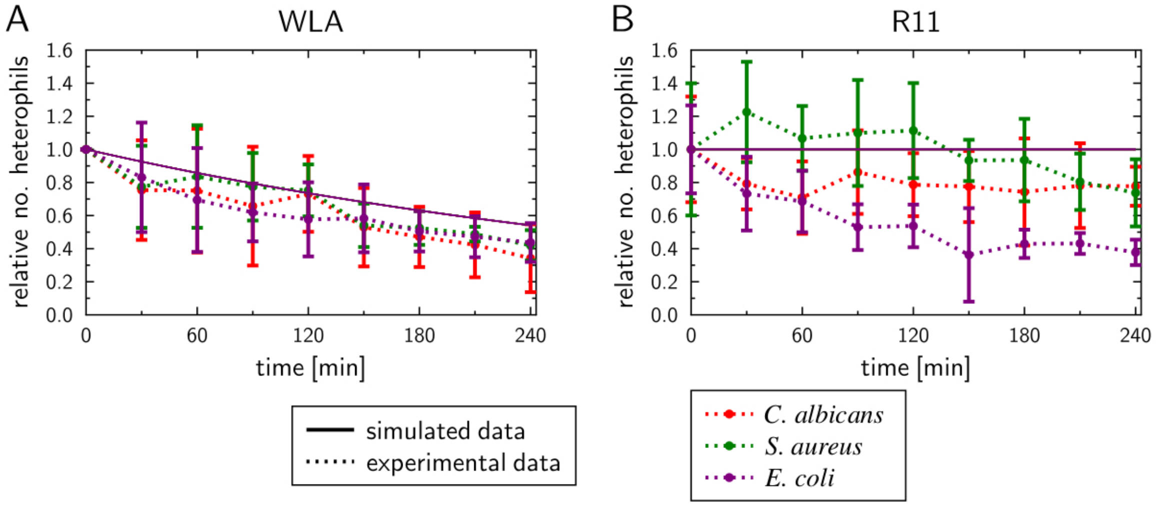

Supplementary Figure 13: Time course of the monocyte number during infection simulated by the MEK-SBM without immune cell killing by extracellular mechanisms induced by pathogens with rate κ_{EM}^{He} for heterophils and rate κ_{EM}^{M} for monocytes. The model was calibrated to experimental data on whole-blood infection of samples from WLA chickens (left column) and R11 chickens (right column). Experimental data are represented by data points that are connected by dotted lines as guide for the eye.

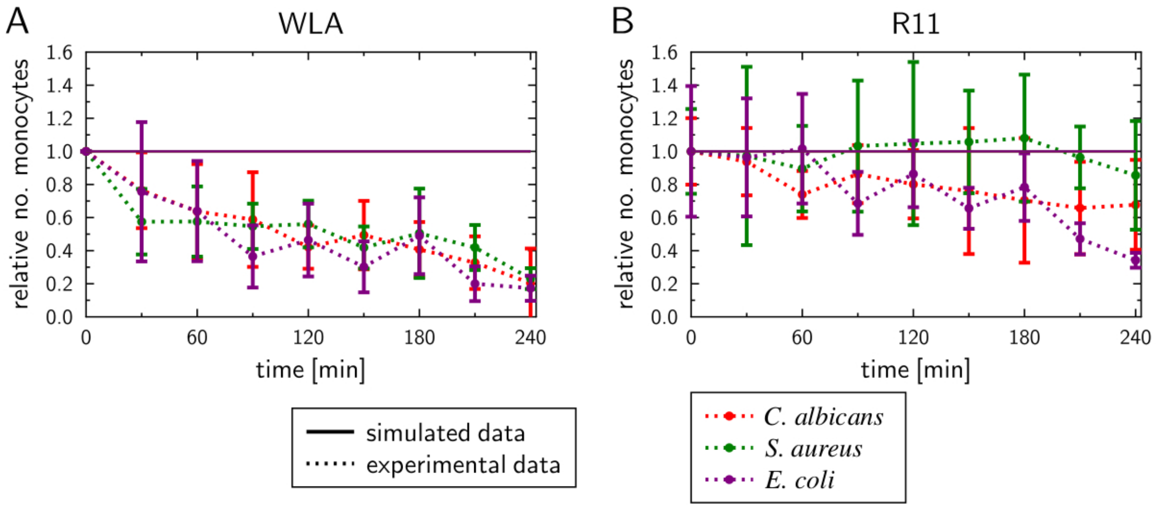

Supplementary Figure 14: Total least squares error (LSE) and corrected Akaike information criterion (AIC_C) of varius SBMs. The LSE scores the agreement between experimental data and simulated data of the SEK-SBM (single extracellular killing mechanism of pathogens), the MEK-SBM (multiple extracellular killing mechanisms of pathogens) and the MEK-SBM with additional immune cell killing mechanism of lysis by pathogens. The data points and error bars depict the mean $\pm$ standard deviation of simulation results observed from 50 simulations for normally distributed transition rates. The model simulations were calibrated to experimental data of whole-blood samples from WLA and R11 chicken that were infected with *C. albicans* (A), *E. coli* (B) and *S. aureus* (C). The corrected AIC (AIC_C) additionally involves the model complexity of the SBMs and is calculated for simulations of whole-blood infection with *C. albicans* (D), *E. coli* (E) and *S. aureus* (F).

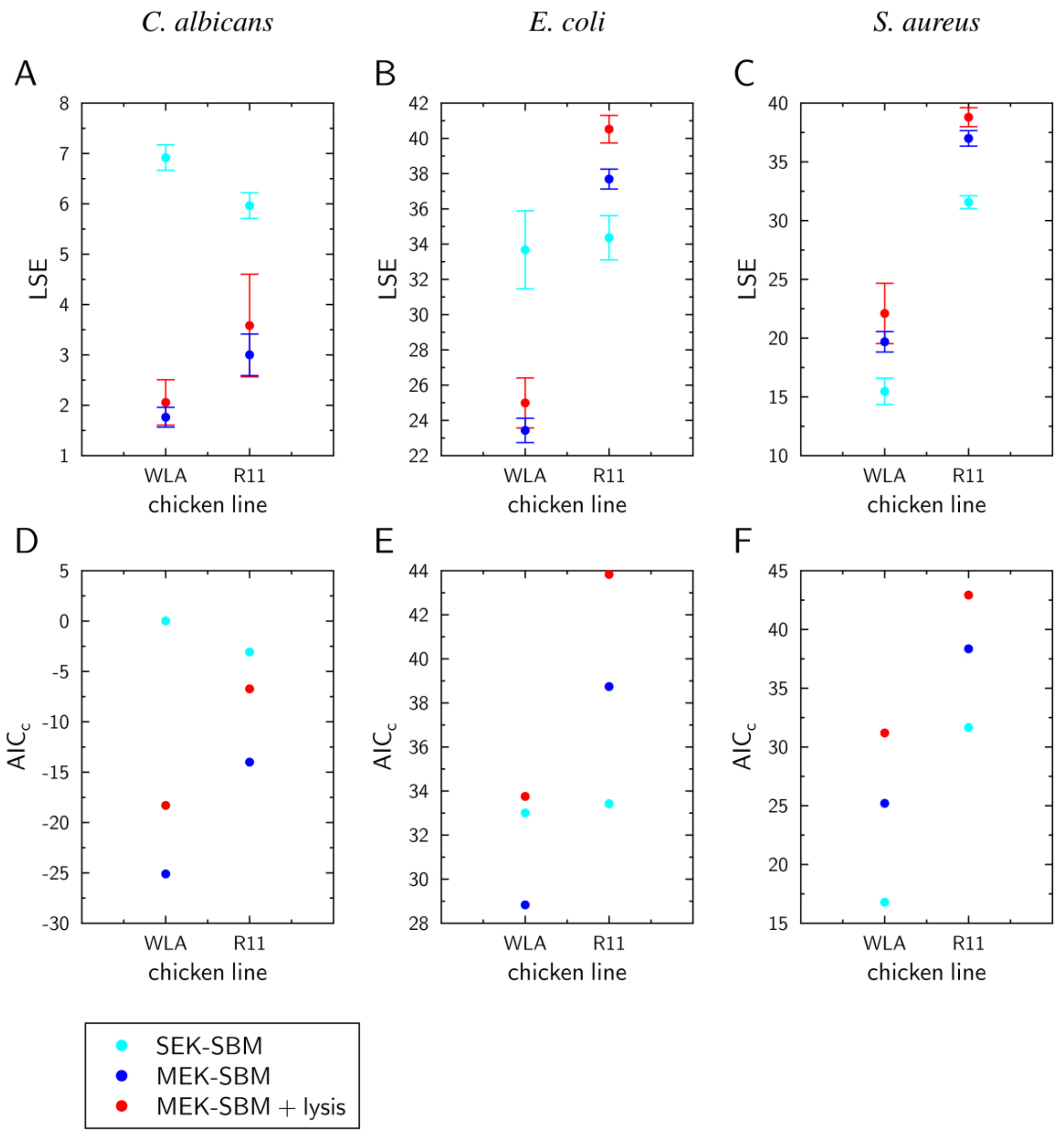

Supplementary Figure 15: Time course of the heterophil number during infection simulated by the MEK-SBM with immune cell killing exclusively by lysis with rate κ_{lysis}^{He} for heterophils and rate κ_{lysis}^{M} for monocytes. The model was calibrated to experimental data on whole-blood infection of samples from WLA chickens (A) and R11 chickens (B). Experimental data are represented by data points that are connected by dashed lines as guide for the eye.

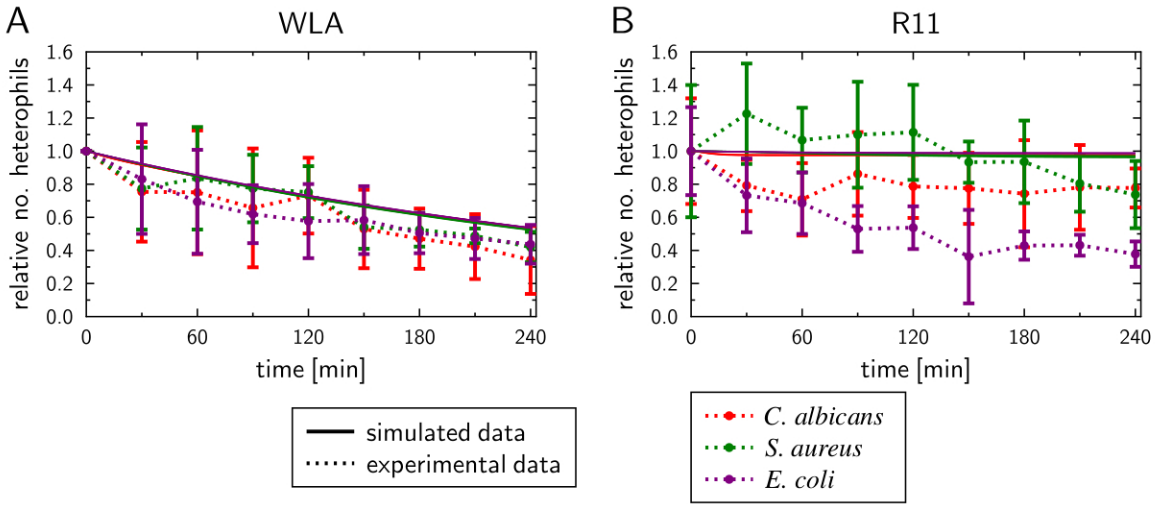

Supplementary Figure 16: Time course of the monocyte number during infection simulated by the MEK-SBM with immune cell killing exclusively by lysis with rate κ_{lysis}^{He} for heterophils and rate κ_{lysis}^{M} for monocytes. The model was calibrated to experimental data on whole-blood infection of samples from WLA chickens (A) and R11 chickens (B). Experimental data are represented by data points that are connected by dashed lines as guide for the eye.

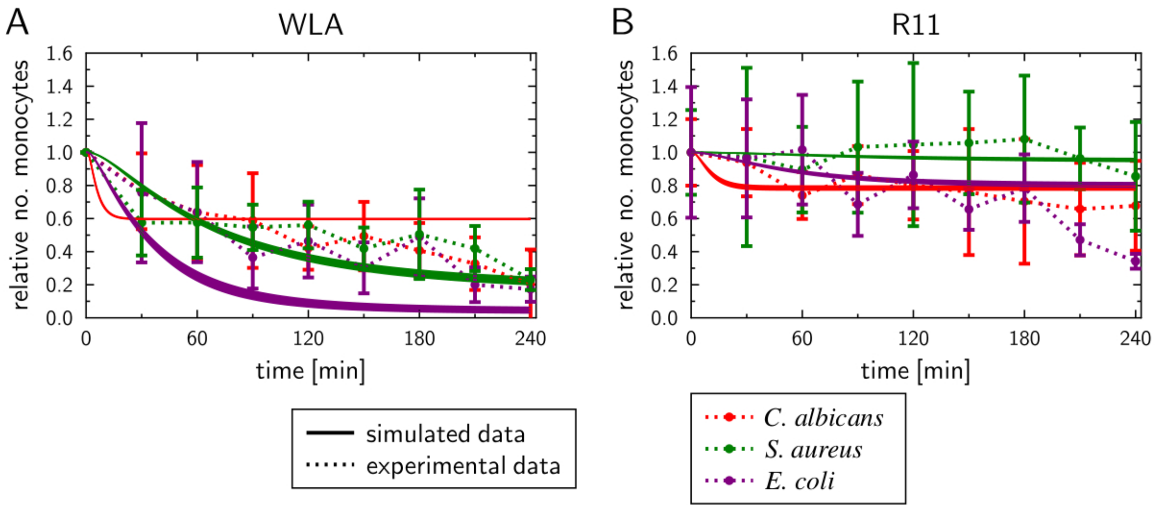

Supplementary Figure 17: Expression of the genes encoding IL-8, K60, MIP1β, and LITAF in infected chicken blood. Gene expression was normalized to GAPDH and expressed as fold change compared to non-infected samples. The graphs represent the fold change of gene expression in infected avian whole blood relative to non-infected blood samples at the respective time points. Data of six independent experiments using blood from different donors is presented as mean and SD.

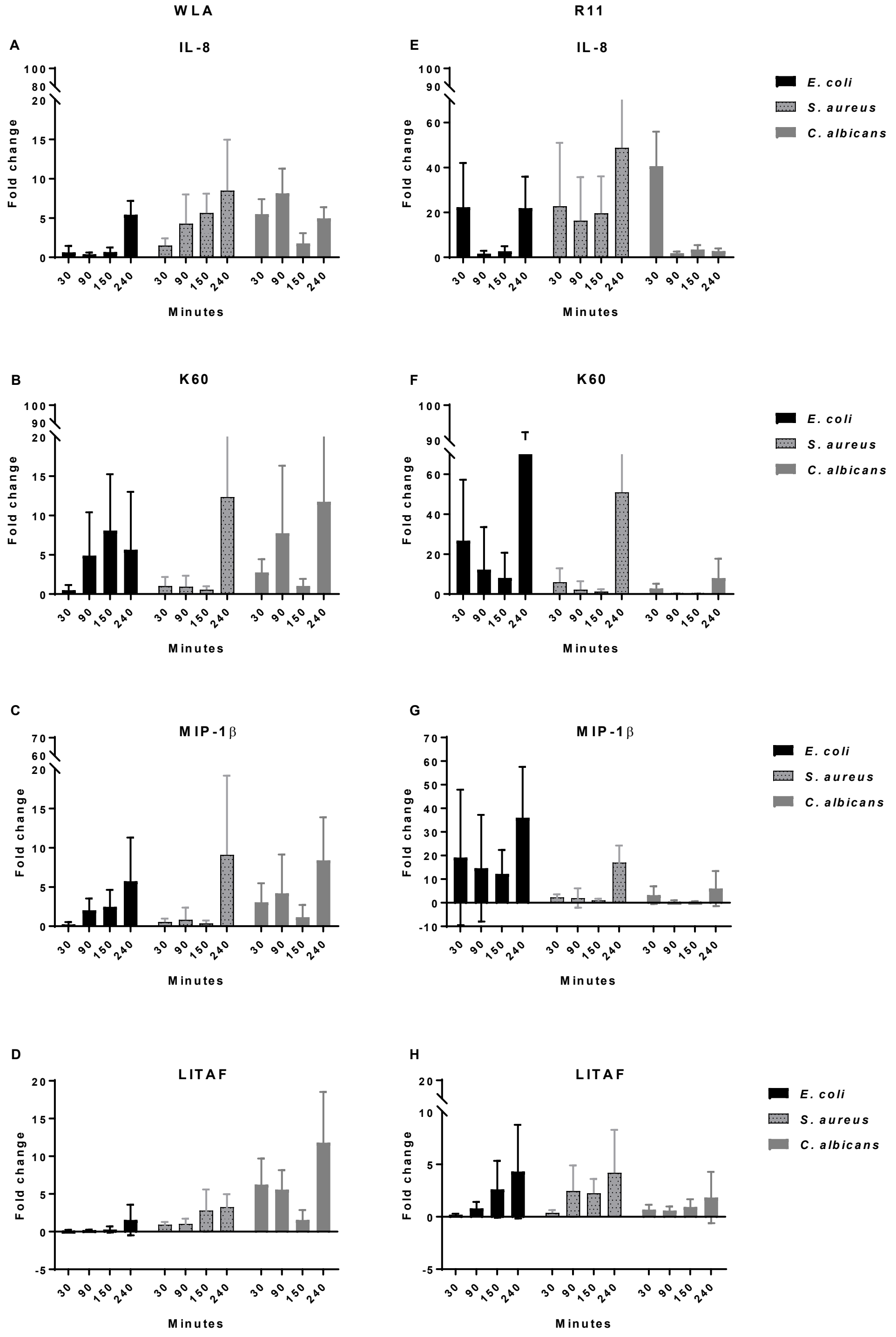

Supplement: Datasheet 2 — Supplementary methods and figures. [file Data_Sheet_2.pdf]
